# Epigenetic silencing of SALL2 confers tamoxifen resistance in breast cancer

**DOI:** 10.15252/emmm.202115618

**Published:** 2022-03-07

**Authors:** Liping Ye, Chuyong Lin, Xi Wang, Qiji Li, Yue Li, Meng Wang, Zekun Zhao, Xianqiu Wu, Dongni Shi, Yunyun Xiao, Liangliang Ren, Yunting Jian, Meisongzhu Yang, Ruizhang Ou, Guangzheng Deng, Ying Ouyang, Xiangfu Chen, Jun Li, Libing Song

The authors became aware that the representative Ki67‐staining image of the ipatasertib tumor had been displayed twice in the figure set. The authors state that the image displayed in Figure 5G, 5G was incorrect and that this correction does not affect the description, interpretation, or conclusions of the manuscript. The authors apologize for this error and any confusion it may have caused.

**Figure 5G:**
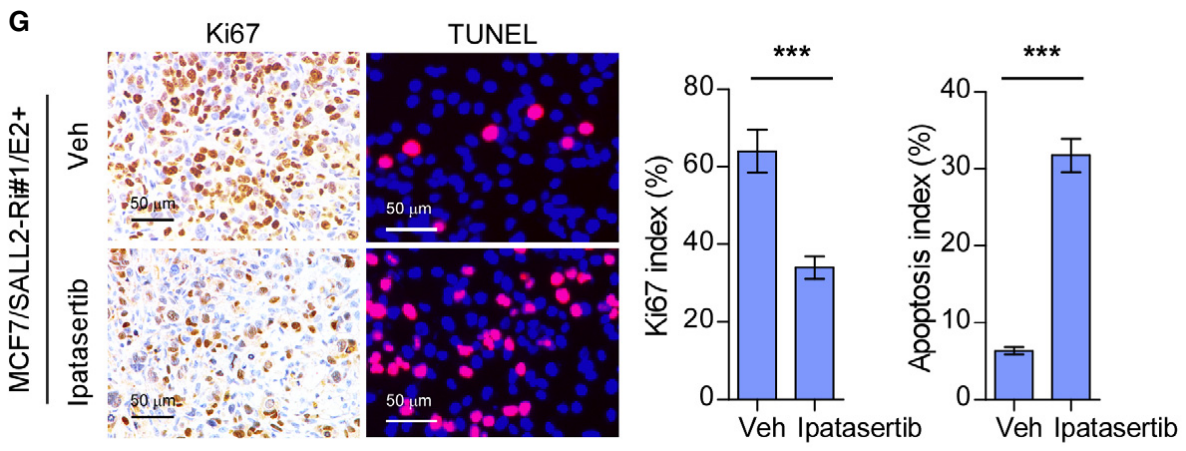
Original

**Figure 5G:**
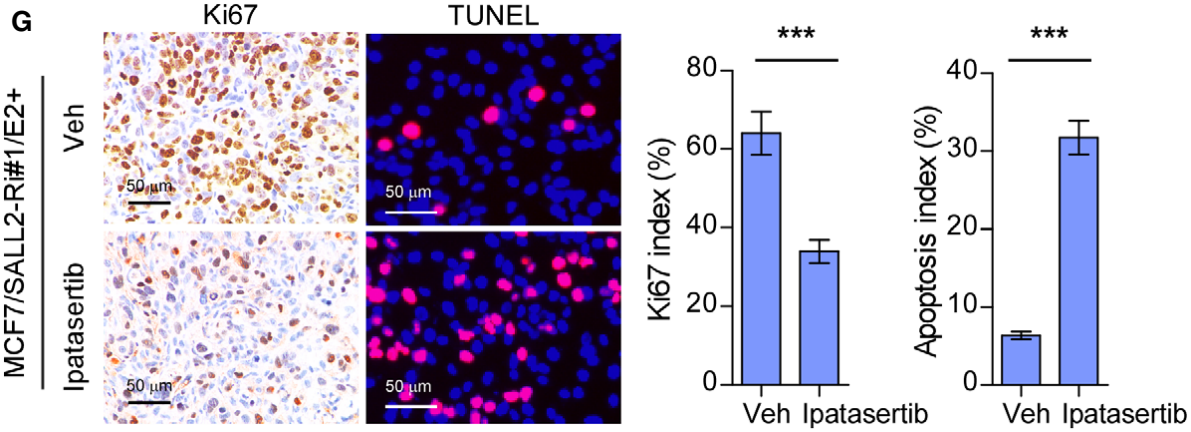
Corrected Source data are available online for this figure.

## Supporting information



Source Data for Figure 5G

