# Supplementary material for: Epigenetic silencing of SALL2 confers tamoxifen resistance in breast cancer
Source: EMBO Mol Med. 2019 Oct 28;11(12):e10638. doi: 10.15252/emmm.201910638 (PMC6895605; doi:10.15252/emmm.201910638)
Supplement: Supplementary file 1 — Appendix [file EMMM-11-e10638-s001.pdf]

Epigenetic silencing of SALL2 confers tamoxifen resistance in breast cancer

Liping Ye, Chuyong Lin, Xi Wang, Qiji Li, Yue Li, Meng Wang, Zekun Zhao, Xianqiu Wu, Dongni Shi, Yunyun Xiao, Liangliang Ren, Yunting Jian, Meisongzhu Yang, Ruizhang Ou, Guangzheng Deng, Ying Ouyang, Xiangfu Chen, Jun Li & Libing Song

APPENDIX

Table of content

Appendix Figures

Appendix Figure S1. SALL2 reduction correlates with poorer clinical outcome of patients with breast cancer.

Appendix Figure S2. Only E1A isoform of SALL2 expresses in breast cancer tissues.

Appendix Figure S3. Validation of the specificity of the SALL2 antibody for IHC.

Appendix Figure S4. SALL2 transcriptionally upregulates PTEN.

Appendix Tables

Appendix Table S1. Clinicopathological characteristics of tamoxifen-treated breast cancer tissues used for RNA-seq analysis.

Appendix Table S2. The correlation between SALL2 expression and clinicopathological characteristics in breast cancer patients (n = 238 cases).

Appendix Table S3. Correlation of SALL2 expression with prognosis of breast cancer patients (n = 238 cases).

Appendix Table S4. Correlation of SALL2 expression with prognosis of tamoxifen-treated patients with ER+ breast cancer (n = 90 cases).

Appendix Table S5. Multivariate analyses of overall survival (OS) and disease free survival (DFS) in breast cancer patients (n = 238 cases).

Appendix Table S6. Multivariate analyses of overall survival (OS) and disease free survival (DFS) in tamoxifen-treated patients with ER+ breast cancer (n = 90 cases).

Appendix Table S7. Primers for real-time PCR analysis.

Appendix Table S8. Primers for BSP assay.

Appendix Table S9. Primers for ChIP-PCR assay.

Appendix Table S10. Summary of exact p-values and number of replicates in figures.

Appendix Figures

Appendix Figure S1

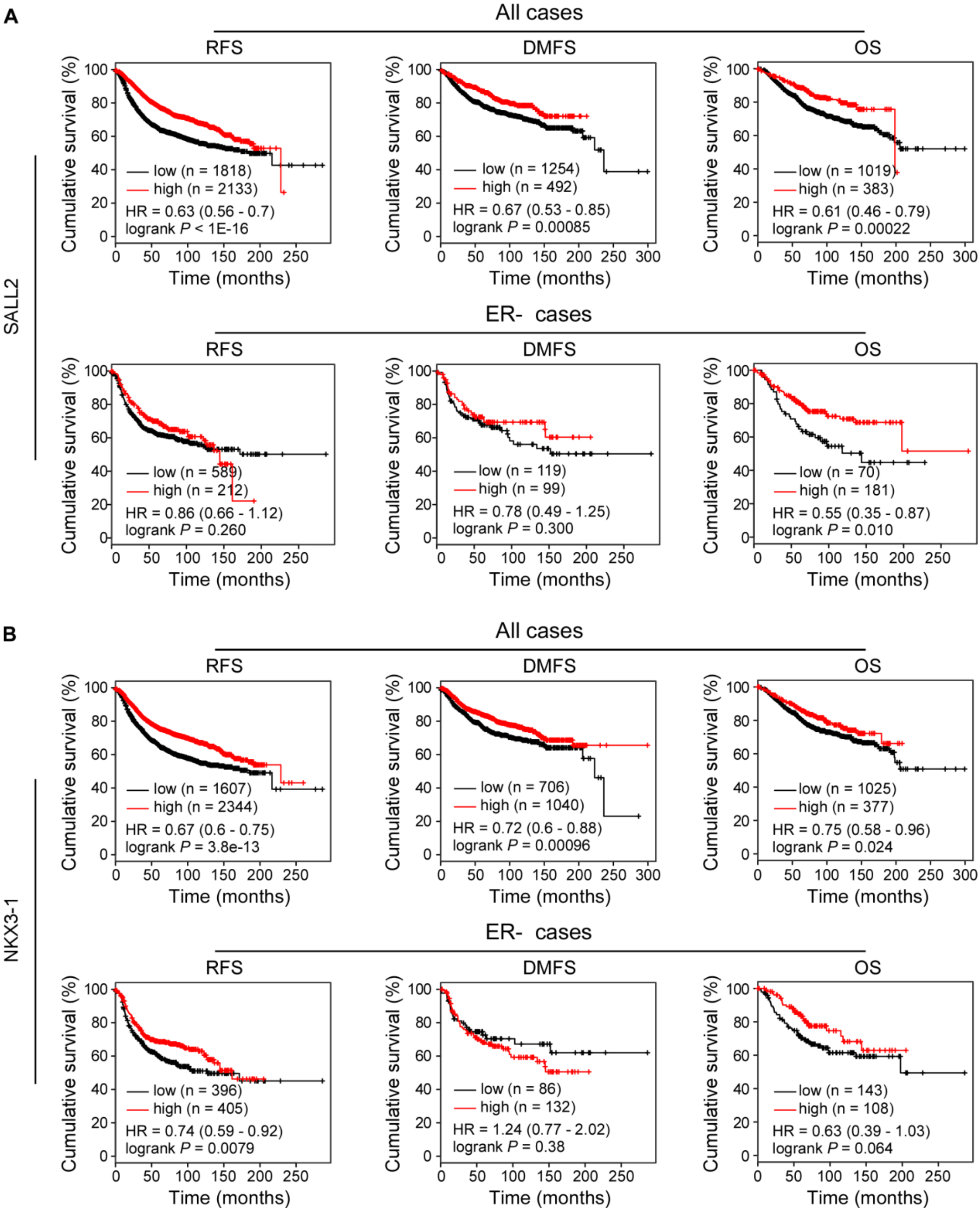

Appendix Figure S1. SALL2 reduction correlates with poorer clinical outcome of patients with breast cancer.

A and **B** Kaplan–Meier (KM) plotter analysis of correlation between SALL2 (A) and NKX3-1 (B) expression with RFS, DMFS, and OS of all patients with breast cancer (upper panel) or patients with ER- breast cancer (lower panel) using the Kaplan-Meier method with the log-rank test (auto select best cutoff).

Appendix Figure S2

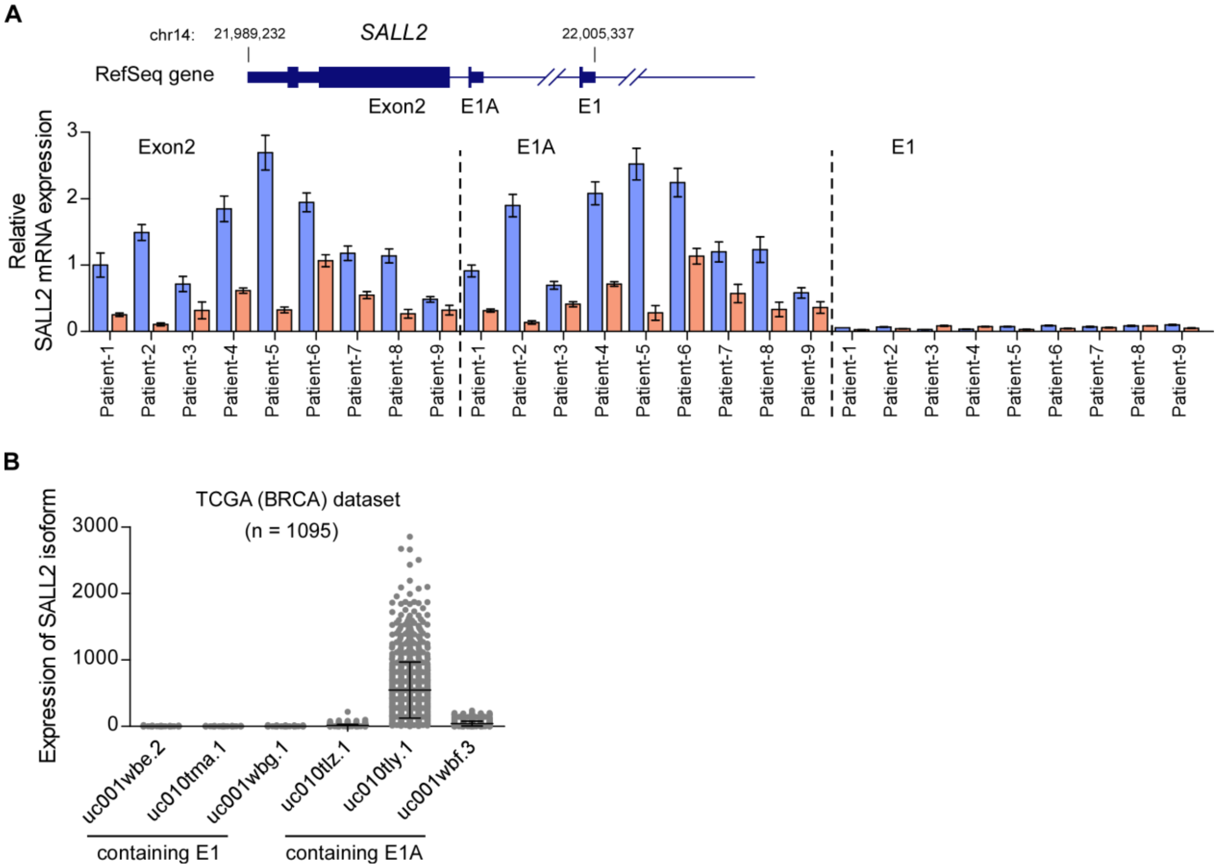

Appendix Figure S2. Only E1A isoform of SALL2 expresses in breast cancer tissues.

A Schematic representation (upper panel) and qRT-PCR analysis (lower panel) of SALL2 gene in the indicated breast cancer tissues using specific PCR primers at alternative exon 1 of E1 or E1A, and the common Exon2. *GAPDH* was used an internal control.

B TCGA dataset analysis of the expression levels of SALL2 isoform, including E1 and E1A, in breast cancer.

Appendix Figure S3

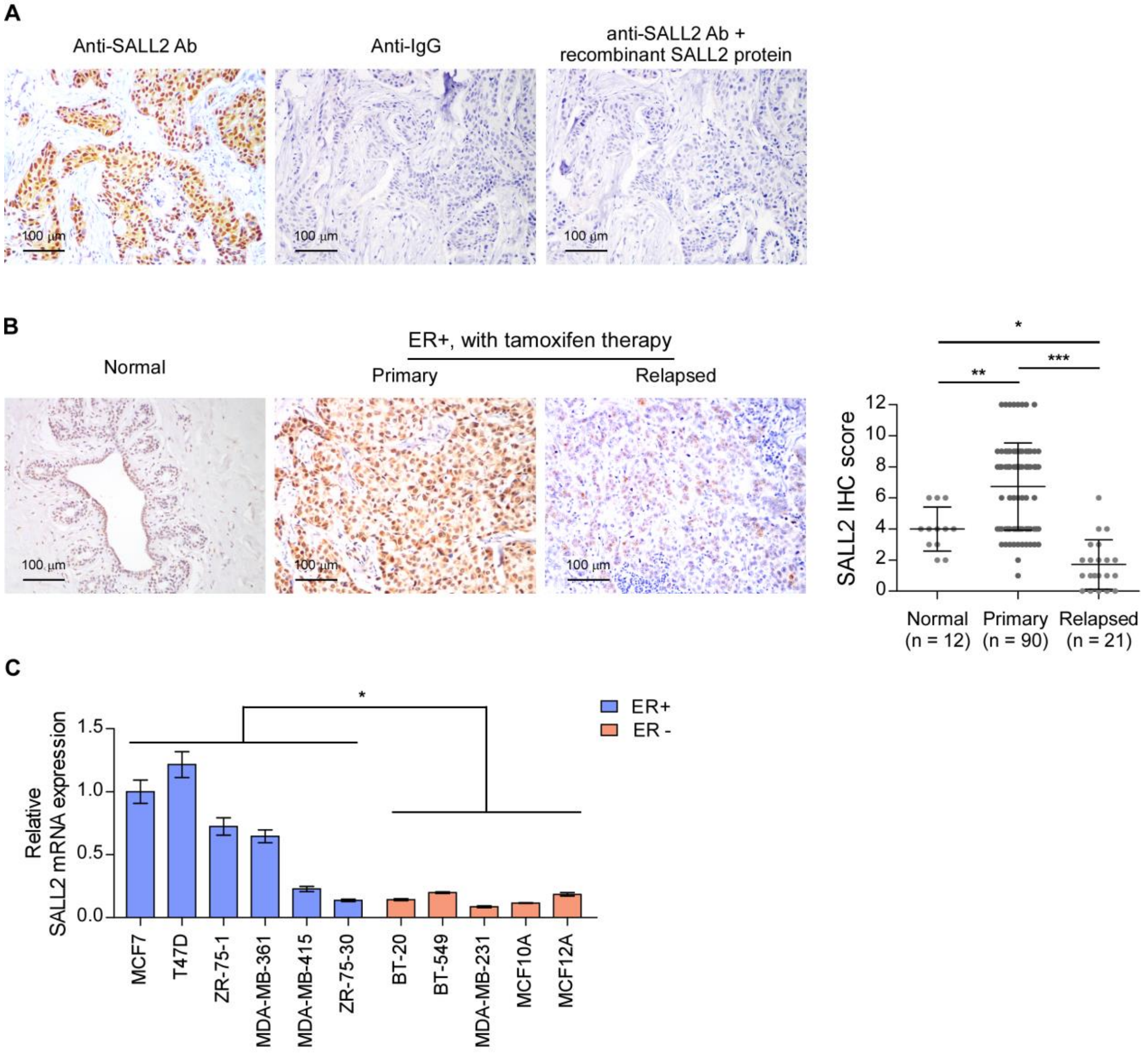

Appendix Figure S3. Validation of the specificity of the SALL2 antibody for IHC.

A Specificity validation of anti-SALL2 antibody via IHC assay using anti-SALL2 antibody, anti-IgG antibody, or anti-SALL2 antibody blocked with recombinant SALL2 protein. Scale bars: 100μm.

B Representative IHC images of SALL2 in normal breast tissues, primary tamoxifen-sensitive ER+ breast cancer tissues, and the relapsed tamoxifen resistant breast cancer tissues. Scale bars: 100μm.

C qRT-PCR analysis of *SALL2* expression in the indicated cell lines. *GAPDH* was used an internal control.

Data information: In (B), data were presented as mean ± SD, *P*-values were determined by one-way ANOVA test. In (C), data were presented as mean ± SD, *P*-values were determined by two-tailed unpaired student's t test. *$P < 0.05$, **$P < 0.01$, ***$P < 0.001$, n.s, no significance. Exact P-values are specified in Appendix Table S10.

Appendix Figure S4

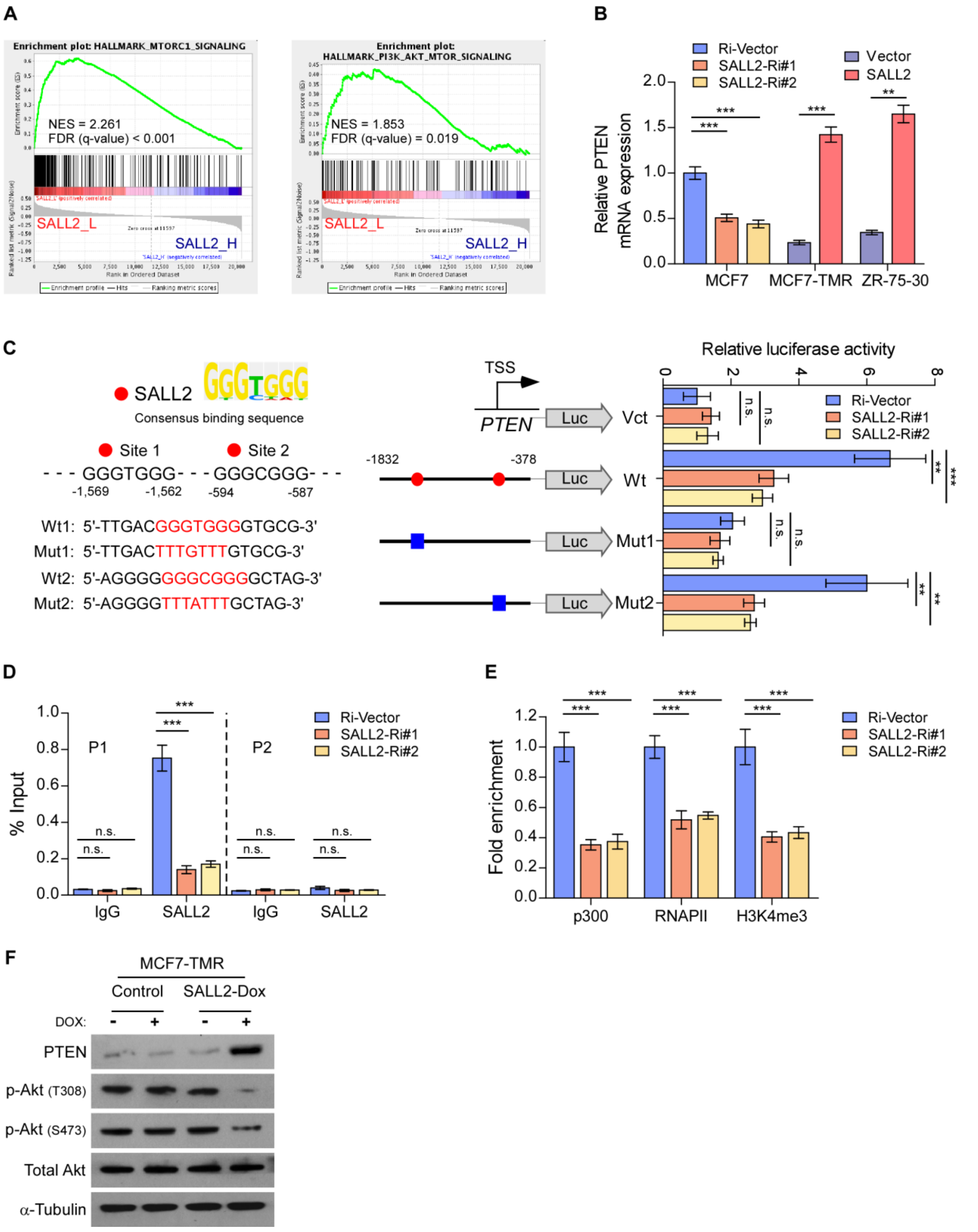

Appendix Figure S4. SALL2 transcriptionally upregulates PTEN.

A GSEA analysis of correlation between SALL2 expression with MTORC1_SIGNALING and PI3K_AKT_MTOR_SIGNALING gene signatures in TCGA (BRCA) dataset. NES, normalized enrichment score; FDR, false discovery rate.

B qRT-PCR analysis of *PTEN* expression in the indicated cell line. *GAPDH* was used an internal control.

C Schematic illustration (upper panel) of the predicted SALL2 binding sites in promoter regions of the PTEN. Quantification of luciferase reporter activity analysis of the PTEN promoter in the indicated cells (lower panel). Putative SALL2-binding sites were shown as red filled circles, and blue filled box showed the mutated SALL2-binding sites. Red letters in each binding region indicate the putative or mutated SALL2-binding sequences. Vct, empty vector; Wt, wild-type; Mut, mutant.

D ChIP analysis of enrichment of SALL2 on the two predicted SALL2 binding sites of the PTEN promoter. IgG was used as a negative control. The red squares represented the qRT-PCR region.

E ChIP analysis of enrichment of p300, RNAP II and H3K4me3 on the PTEN promoter.

F WB analysis of the indicated protein expression in the MCF7-TMR/control and MCF7-TMR/SALL2-Dox cells treated with or without doxycycline (Dox); α-tubulin was used as a loading control.

Data information: In (B, left; C-E), *P*-values were determined by one-way ANOVA test. In (B, middle and right), data are presented as mean ± SD, *P*-values were determined by two-tailed student's t test. $^{*}P < 0.05$, $^{**}P < 0.01$, $^{***}P < 0.001$, n.s, no significance. Exact P-values are specified in Appendix Table S10.

Appendix Tables

Appendix Table S1. Clinicopathological characteristics of tamoxifen-treated breast cancer tissues used for RNA-seq analysis

Patient No.	ER	PR	Her2	Days from treatment to relapse	Site of relapse
Patient-1	+++	+++	-	1288	BN
Patient-2	+++	+++	++	686	BN
Patient-3	+++	++	-	756	CW
Patient-4	++	++	-	660	BR
Patient-5	++	++	+	392	OV
Patient-6	+++	+++	-	1096	LI
Patient-7	+	+++	+++	1726	CW
Patient-8	++	++	-	1191	LI
Patient-9	+	+	-	165	LU

BN, bone; BR, brain; CW, chest wall; LU, lung; LI, liver; OV, ovary.

Appendix Table S2. The correlation between SALL2 expression and clinicopathological characteristics in breast cancer patients (n = 238 cases)

Characteristics	Number (%)	SALL2 expression		P values
		Low, no. cases (%)	High, no. cases (%)	
**Gender**				
Female	238 (100)	116 (48.7)	122 (51.3)	-
**Age (years)**				
< 50	137 (57.6)	66 (27.7)	71 (29.8)	0.839
≥ 50	101 (42.4)	50 (21.0)	51 (21.4)	
**T stage**				
1	75 (31.5)	32 (13.4)	43 (18.1)	0.021
2	132 (55.5)	61 (25.6)	71 (29.8)	
3	22 (9.2)	17 (7.1)	5 (2.1)	
4	9 (3.8)	6 (2.5)	3 (1.3)	
**N stage**				
0	102 (42.9)	33 (13.9)	69 (29.0)	< 0.001
1	65 (27.3)	35 (14.7)	30 (12.6)	
2	38 (16.0)	27 (11.3)	11 (4.6)	
3	33 (13.9)	21 (8.8)	12 (5.0)	
**Clinical stage**				
I	39 (16.4)	11 (4.6)	28 (11.8)	< 0.001
II	116 (48.7)	50 (21.0)	66 (27.7)	
III	83 (34.9)	55 (23.1)	28 (11.8)	
**ER expression**				

Negative	74 (31.1)	53 (22.3)	21 (8.8)	< 0.001
Positive	164 (68.9)	63 (26.5)	101 (42.4)	
**PR expression**				
Negative	81 (34.0)	47 (19.7)	34 (14.3)	0.040
Positive	157 (66.0)	69 (29.0)	88 (37.0)	
**HER2 expression**				
Negative	193 (81.1)	89 (37.4)	104 (43.7)	0.093
Positive	45 (18.9)	27 (11.3)	18 (7.6)	
**Ki67 expression**				
Low	98 (41.2)	37 (15.5)	61 (25.6)	0.005
High	140 (58.8)	79 (33.2)	61 (25.6)	
**Endocrine therapy**				
With tamoxifen	90 (37.8)	33 (13.9)	57 (23.9)	< 0.001
Other without tamoxifen	74 (31.1)	30 (12.6)	44 (18.5)	
None	74 (31.1)	53 (22.3)	21 (8.8)	

Appendix Table S3. Correlation of SALL2 expression with prognosis of breast cancer patients (n = 238 cases)

Characteristics	Number (%)	SALL2 expression		P values
		Low, No. cases (%)	High, No. cases (%)	
**Living status**				
Living	171 (71.8)	67 (28.2)	104 (43.7)	< 0.001
Death	67 (28.2)	49 (20.6)	18 (7.6)	
**Relapse/Metastasis status**				
Negative	160 (67.2)	62 (26.1)	98 (41.2)	< 0.001
Positive	78 (32.8)	54 (22.7)	24 (10.1)	

Appendix Table S4. Correlation of SALL2 expression with prognosis of tamoxifen-treated patients with ER+ breast cancer (n = 90 cases)

Characteristics	Number (%)	SALL2 expression		*P* values
		Low, no. cases (%)	High, no. cases (%)	
**Living status**				
Living	72 (80.0)	21 (23.3)	51 (56.7)	0.003
Death	18 (20.0)	12 (13.3)	6 (6.7)	
**Relapse/Metastasis status**				
Negative	69 (76.7)	21 (23.3)	48 (53.3)	0.026
Positive	21 (23.3)	12 (13.3)	9 (10.0)	

Appendix Table S5. Multivariate analyses of overall survival (OS) and disease free survival (DFS) in breast cancer patients (n = 238 cases)

Characteristics	Overall Survival		Disease-Free Survival	
	HR (95% CI)	P values	HR (95% CI)	P values
**Age** (≥ 50 years)	1.038 (0.619-1.743)	0.886	0.972 (0.596-1.586)	0.911
**T stage** (2-4)	1.447 (0.770-2.720)	0.250	1.374 (0.779-2.425)	0.273
**N stage** (1-3)	1.428 (0.676-3.016)	0.350	1.913 (0.959-3.812)	0.065
**Clinical stage** (III)	2.645 (1.484-4.715)	0.001	2.336 (1.385-3.938)	0.001
**ER (+)**	0.396 (0.199-0.789)	0.008	0.191 (0.098-0.373)	<0.001
**PR (+)**	0.469 (0.235-0.934)	0.031	0.190 (0.093-0.388)	<0.001
**HER2 (+)**	1.191 (0.658-2.157)	0.563	1.441 (0.841-2.469)	0.184
**Ki67 expression** (high)	1.241 (0.699-2.202)	0.461	1.267 (0.760-2.114)	0.364
**SALL2 expression** (low)	2.220 (1.251-3.940)	0.006	1.939 (1.167-3.222)	0.011

HR, hazard ratio; CI, confidence interval.

Appendix Table S6. Multivariate analyses of overall survival (OS) and disease free survival (DFS) in tamoxifen-treated patients with ER+ breast cancer (n = 90 cases)

Characteristics	Overall Survival		Disease-Free Survival	
	HR (95% CI)	P values	HR (95% CI)	P values
**Age** (≥ 50 years)	1.017 (0.329-3.140)	0.977	1.464 (0.552-3.879)	0.443
**T stage** (2-4)	2.351 (0.731-7.560)	0.151	1.427 (0.540-3.770)	0.473
**N stage** (1-3)	3.037 (0.818-11.277)	0.097	5.520 (1.553-19.618)	0.008
**Clinical stage** (III)	1.235 (0.430-3.551)	0.695	0.961 (0.365-2.532)	0.936
**Ki67 expression** (high)	1.019 (0.393-2.642)	0.969	1.159 (0.485-2.767)	0.740
**SALL2 expression** (low)	4.408 (1.559-12.466)	0.005	2.811 (1.126-7.018)	0.027

HR, hazard ratio; CI, confidence interval.

Appendix Table S7. Primers for real-time PCR analysis

Gene		Sequence(5'-3')
SALL2	forward	AGGTGCCGGTACTGAAGATG
	reverse	CACTGACCAGCCAAAACCTT
ESR1	forward	GGCTACATCATCTCGGTTCC
	reverse	TCCAGAGACTTCAGGGTGCT
TFF1	forward	CCAGTGTGCAAATAAGGGCTGC
	reverse	AGGCAGATCCCTGCAGAAGTGT
PGR	forward	GTCGCCTTAGAAAGTGCTGTCAG
	reverse	GCTTGGCTTTCATTTGGAACGCC
PTEN	forward	TGAGTTCCCTCAGCCGTTACCT
	reverse	GAGGTTTCCTCTGGTCCTGGTA
CA12	forward	AGGCCAGGAAGCATTCGTC
	reverse	GGGAAGGGTCGTCCATGTG
RET	forward	GTGTCTTCGATGCAGACGTG
	reverse	CATGGTGCGGTTCTCCGAG
STC2	forward	ACAGGTTCGGCTGCATAAGC
	reverse	GAGGTCCACGTAGGGTTCG
KAAG1	forward	ACCTGCTACGGGCAGAATCA
	reverse	TCCTCTCTCCTGCCCTCACTA
PMEPA1	forward	TGTCAGGCAACGGAATCCC
	reverse	CAGGTACGGATAGGTGGGC
MAST4	forward	GATGGTGAGGCGGAGCAAGAAA
	reverse	CAAGCAGGAGAAACTTCGGCTG
MSX2	forward	CACCCTGAGGAAACACAAGAC
	reverse	TGCACGCTCTGCAATGGAG
GFRA1	forward	GACTCCTGCAAGACGAATTACA
	reverse	GCTGCTGACAGACCTTGACT

SALL2-E1A	forward	CAGACTGCGGAGATGGAGATC
	reverse	CGCTTTCTCACTCCAGCTTCT
SALL2-E1	forward	CGCTCACTTGGTCTTAACCG
	reverse	TCTGTGTCTCTGGGAAAGAAGAAC
SALL2-E2	forward	TGCTACTTCTTGTCTATGATGGGC
	reverse	CAGGGAAGCCAAGGAGAAGAC
GAPDH	forward	AAGGTGAAGGTCGGAGTCAA
	reverse	AATGAAGGGGTCATTGATGG

Appendix Table S8. Primers for BSP assay

Gene (promoter)		Sequence(5'-3')
SALL2 promoter	forward	ATGGTTGTGGGGGAAGTGGA
	reverse	TCAACTCCTCCCAAATCTCC
ESR1 promoter	forward	ATATTAAAGTATTTGGGATGG
	reverse	CTCCAAATAATAAAACACCTACTAAC

Appendix Table S9. Primers for ChIP-PCR assay

Gene (promoter)		Sequence(5'-3')
ESR1-P1	forward	CTGGATGCATGTTTAAGGTAGCT
	reverse	ACACATAGGGGGCTCTGGAC
ESR1-P2	forward	GCCAGTCTATAGTTTGGCTGATC
	reverse	CCTGACCACTCACTCACTATCG
PTEN-P1	forward	TGACTCAAATTGTCGTCTGTAGTTC
	reverse	GACCTGTCACCGTGAGAGAGC
PTEN-P2	forward	CTTCCTACCGTTCCGTACTTTC
	reverse	CCAAGAGAGTCGAGCATCTTTC

Appendix Table S10. Summary of exact *P*-values and number of replicates in figures

Figure		Exact *P*-value	n-value in each group
1	E	MCF7: Ri-Vector vs. SALL2-Ri#1: *P* = 0.0003; Ri-Vector vs. SALL2-Ri#2: *P* < 0.0001	3
		T47D: Ri-Vector vs. SALL2-Ri#1: *P* = 0.0005; Ri-Vector vs. SALL2-Ri#2: *P* = 0.0010	
		ZR-75-1: Ri-Vector vs. SALL2-Ri#1: *P* = 0.0002; Ri-Vector vs. SALL2-Ri#2: *P* = 0.0007	
		MCF7: Ri-Vector vs. NKX3-1-Ri#1: *P* = 0.0071; Ri-Vector vs. NKX3-1-Ri#2: *P* = 0.0167	
		T47D: Ri-Vector vs. NKX3-1-Ri#1: *P* = 0.0129; Ri-Vector vs. NKX3-1-Ri#2: *P* = 0.1126	
		ZR-75-1: Ri-Vector vs. NKX3-1-Ri#1: *P* = 0.0103; Ri-Vector vs. NKX3-1-Ri#2: *P* = 0.0023	
3	A	MCF7: Ri-Vector vs. SALL2-Ri#1: *P* < 0.0001; Ri-Vector vs. SALL2-Ri#2: *P* < 0.0001	3
		MCF7-TMR: Vector vs. SALL2: *P* = 0.0009; ZR-75-30: Vector vs. SALL2: *P* = 0.0007	
	C	Vct: Ri-Vector vs. SALL2-Ri#1: *P* = 0.2200; Ri-Vector vs. SALL2-Ri#2: *P* = 0.0545	3
		Wt: Ri-Vector vs. SALL2-Ri#1: *P* = 0.0005; Ri-Vector vs. SALL2-Ri#2: *P* = 0.0008	
		Mut: Ri-Vector vs. SALL2-Ri#1: *P* = 0.5332; Ri-Vector vs. SALL2-Ri#2: *P* = 0.2295	
	D	P1-SALL2: Ri-Vector vs. SALL2-Ri#1: *P* = 0.0581; Ri-Vector vs. SALL2-Ri#2: *P* = 0.2707	3
		P1-IgG: Ri-Vector vs. SALL2-Ri#1: *P* = 0.2818; Ri-Vector vs. SALL2-Ri#2: *P* = 0.1812	
		P2-SALL2: Ri-Vector vs. SALL2-Ri#1: *P* < 0.0001; Ri-Vector vs. SALL2-Ri#2: *P* < 0.0001	
		P2-IgG: Ri-Vector vs. SALL2-Ri#1: *P* = 0.3520; Ri-Vector vs. SALL2-Ri#2: *P* = 0.0626	
	E	p300: Ri-Vector vs. SALL2-Ri#1: *P* < 0.0001; Ri-Vector vs. SALL2-Ri#2: *P* < 0.0001	3
		RNAPII: Ri-Vector vs. SALL2-Ri#1: *P* < 0.0001; Ri-Vector	

	vs. SALL2-Ri#2: $P < 0.0001$<br>H3K4me3: Ri-Vector vs. SALL2-Ri#1: $P < 0.0001$; Ri-Vector vs. SALL2-Ri#2: $P < 0.0001$	
F	MCF7/Ri-Vector: Vehicle vs. E2: $P < 0.0001$; Vehicle vs. TAM: $P = 0.0490$<br>MCF7/SALL2-Ri#1: Vehicle vs. E2: $P = 0.9776$; Vehicle vs. TAM: $P = 0.9946$<br>MCF7-TMR/Ri-Vector: Vehicle vs. E2: $P = 0.5664$; Vehicle vs. TAM: $P = 0.9950$<br>MCF7-TMR/SALL2-Ri#1: Vehicle vs. E2: $P < 0.0001$; Vehicle vs. TAM: $P = 0.0098$<br>ZR-75-30/Ri-Vector: Vehicle vs. E2: $P = 0.3135$; Vehicle vs. TAM: $P = 0.8470$<br>ZR-75-30/SALL2-Ri#1: Vehicle vs. E2: $P < 0.0001$; Vehicle vs. TAM: $P = 0.0446$	3
G	TFF1: MCF7/Ri-Vector: Vehicle vs. E2: $P < 0.0001$; Vehicle vs. TAM: $P = 0.0331$<br>PGR: MCF7/Ri-Vector: Vehicle vs. E2: $P < 0.0001$; Vehicle vs. TAM: $P = 0.0394$<br>TFF1: MCF7/SALL2-Ri#1: Vehicle vs. E2: $P = 0.2653$; Vehicle vs. TAM: $P = 0.7260$<br>PGR: MCF7/SALL2-Ri#1: Vehicle vs. E2: $P = 0.2589$; Vehicle vs. TAM: $P = 0.7573$<br>TFF1: MCF7-TMR/Vector: Vehicle vs. E2: $P = 0.2691$; Vehicle vs. TAM: $P = 0.9536$<br>PGR: MCF7-TMR/Vector: Vehicle vs. E2: $P = 0.9369$; Vehicle vs. TAM: $P = 0.8193$<br>TFF1: MCF7-TMR/SALL2: Vehicle vs. E2: $P < 0.0001$; Vehicle vs. TAM: $P = 0.0091$<br>PGR: MCF7-TMR/SALL2: Vehicle vs. E2: $P < 0.0001$; Vehicle vs. TAM: $P = 0.0009$<br>TFF1: ZR-75-30/Vector: Vehicle vs. E2: $P = 0.0934$; Vehicle vs. TAM: $P = 0.9874$<br>PGR: ZR-75-30/Vector: Vehicle vs. E2: $P = 0.8475$; Vehicle vs. TAM: $P = 0.4513$<br>TFF1: ZR-75-30/SALL2: Vehicle vs. E2: $P < 0.0001$; Vehicle vs. TAM: $P = 0.0002$	3

		PGR: ZR-75-30/SALL2: Vehicle vs. E2: *P* < 0.0001; Vehicle vs. TAM: *P* = 0.0004	
4	A	E2: Ri-Vector vs. SALL2-Ri#1: *P* < 0.0001; E2+TAM: Ri-Vector vs. SALL2-Ri#1: *P* < 0.0001	8
	B	E2: Ri-Vector vs. SALL2-Ri#1: *P* < 0.0001; E2+TAM: Ri-Vector vs. SALL2-Ri#1: *P* < 0.0001	8
	D	Ri-Vector without E2 vs. SALL2-Ri#1 without E2: *P* < 0.0001	8
	G	CA12: Vehicle: Control vs. SALL2-Dox: *P* = 0.6639; DOX: Control vs. SALL2-Dox: *P* < 0.0001<br>RET: Vehicle: Control vs. SALL2-Dox: *P* = 0.8277; DOX: Control vs. SALL2-Dox: *P* < 0.0001<br>STC2: Vehicle: Control vs. SALL2-Dox: *P* = 0.9745; DOX: Control vs. SALL2-Dox: *P* < 0.0001<br>KAAG1: Vehicle: Control vs. SALL2-Dox: *P* = 0.9825; DOX: Control vs. SALL2-Dox: *P* < 0.0001<br>PMEPA1: Vehicle: Control vs. SALL2-Dox: *P* = 0.9948; DOX: Control vs. SALL2-Dox: *P* < 0.0001<br>MAST4: Vehicle: Control vs. SALL2-Dox: *P* = 0.9987; DOX: Control vs. SALL2-Dox: *P* < 0.0001<br>MSX2: Vehicle: Control vs. SALL2-Dox: *P* = 0.9910; DOX: Control vs. SALL2-Dox: *P* < 0.0001<br>GFRA1: Vehicle: Control vs. SALL2-Dox: *P* = 0.9986; DOX: Control vs. SALL2-Dox: *P* < 0.0001	3
	H	Control: Vehicle vs. DOX: *P* = 0.8833; Vehicle vs. TAM: *P* = 0.9903; Vehicle vs. DOX+TAM: *P* = 0.1981<br>SALL2-Dox: Vehicle vs. DOX: *P* < 0.0001; Vehicle vs. TAM: *P* = 0.3135; Vehicle vs. DOX+TAM: *P* < 0.0001	3
	I	MCF7-TMR/SALL2-Dox: TAM-: DOX- vs. DOX+: *P* < 0.0001; TAM+: DOX- vs. DOX+: *P* < 0.0001	8
5	D	Left panel: Vector vs. SALL2: *P* = 0.0005; SALL2 vs. SALL2+SF1670: *P* = 0.0026<br>Right panel: Vector vs. SALL2: *P* < 0.0001; SALL2 vs. SALL2+SF1670: *P* < 0.0001	3
	E	E2+: Ipatasertib- vs. Ipatasertib+: *P* < 0.0001; E2-: Ipatasertib- vs. Ipatasertib+: *P* < 0.0001	8
	F	E2+: Ipatasertib- vs. Ipatasertib+: *P* < 0.0001; E2-: Ipatasertib-	8

		vs. Ipatasertib+: $P < 0.0001$	
	G	Ki67: Vehicle vs. Ipatasertib: $P < 0.0001$; Apoptosis: Vehicle vs. Ipatasertib: $P < 0.0001$	8
6	B	MCF7: Vehicle vs. 5-Aza-dC (2μM): $P = 0.3208$; Vehicle vs. 5-Aza-dC (5μM): $P = 0.2380$<br>MCF7-TAM (24h): Vehicle vs. 5-Aza-dC (2μM): $P = 0.1076$; Vehicle vs. 5-Aza-dC (5μM): $P = 0.0553$<br>MCF7-TMR: Vehicle vs. 5-Aza-dC (2μM): $P = 0.0012$; Vehicle vs. 5-Aza-dC (5μM): $P < 0.0001$	3
	C	MCF7 vs. MCF7-TAM (24h): $P = 0.7968$; MCF7 vs. MCF7-TMR: $P = 0.0002$; MCF7-TAM (24h) vs. MCF7-TMR: $P = 0.0003$	3
	D	5mc: MCF7 vs. MCF7-TAM (24h): $P = 0.7211$; MCF7 vs. MCF7-TMR: $P < 0.0001$; MCF7-TAM (24h) vs. MCF7-TMR: $P < 0.0001$<br>IgG: MCF7 vs. MCF7-TAM (24h): $P = 0.4099$; MCF7 vs. MCF7-TMR: $P = 0.9998$; MCF7-TAM (24h) vs. MCF7-TMR: $P = 0.4014$	3
	E	DNMT1: MCF7 vs. MCF7-TAM (24h): $P = 0.0990$; MCF7 vs. MCF7-TMR: $P < 0.0001$; MCF7-TAM (24h) vs. MCF7-TMR: $P < 0.0001$<br>DNMT3A: MCF7 vs. MCF7-TAM (24h): $P = 0.0514$; MCF7 vs. MCF7-TMR: $P = 0.2775$; MCF7-TAM (24h) vs. MCF7-TMR: $P = 0.4269$<br>DNMT3B: MCF7 vs. MCF7-TAM (24h): $P = 0.0147$; MCF7 vs. MCF7-TMR: $P < 0.0001$; MCF7-TAM (24h) vs. MCF7-TMR: $P < 0.0001$<br>H3K27me3: MCF7 vs. MCF7-TAM (24h): $P = 0.9654$; MCF7 vs. MCF7-TMR: $P = 0.0006$; MCF7-TAM (24h) vs. MCF7-TMR: $P = 0.0005$<br>H3K9me2: MCF7 vs. MCF7-TAM (24h): $P = 0.6527$; MCF7 vs. MCF7-TMR: $P < 0.0001$; MCF7-TAM (24h) vs. MCF7-TMR: $P < 0.0001$<br>H3K4me3: MCF7 vs. MCF7-TAM (24h): $P = 0.8169$; MCF7 vs. MCF7-TMR: $P = 0.0012$; MCF7-TAM (24h) vs. MCF7-TMR: $P = 0.0007$	3
7	A	TAM-: 5-Aza-dC- vs. 5-Aza-dC+: $P < 0.0001$; TAM+:	8

		5-Aza-dC- vs. 5-Aza-dC+: *P* < 0.0001	
	B	TAM-: 5-Aza-dC- vs. 5-Aza-dC+: *P* < 0.0001; TAM+: 5-Aza-dC- vs. 5-Aza-dC+: *P* < 0.0001	8
	C	Vehicle vs. 5-Aza-dC: *P* < 0.0001; Vehicle vs. TAM: *P* = 0.3545; Vehicle vs. 5-Aza-dC+TAM: *P* < 0.0001	8
	D	Vehicle vs. 5-Aza-dC: *P* < 0.0001; Vehicle vs. TAM: *P* = 0.2792; Vehicle vs. 5-Aza-dC+TAM: *P* < 0.0001	8
	G	TAM-: 5-Aza-dC- vs. 5-Aza-dC+: *P* = 0.7257; TAM+: 5-Aza-dC- vs. 5-Aza-dC+: *P* = 0.6940	8
	H	Ki67: Vehicle vs. 5-Aza-dC: *P* = 0.7720; Vehicle vs. TAM: *P* = 0.9485; Vehicle vs. 5-Aza-dC+TAM: *P* = 0.2222<br>Apoptosis: Vehicle vs. 5-Aza-dC: *P* = 0.9449; Vehicle vs. TAM: *P* = 0.6450; Vehicle vs. 5-Aza-dC+TAM: *P* = 0.9986	8
EV1	A	Primary vs. Relapsed: *P* = 0.0061	9
	B	Patient-1: Primary vs. Relapsed: *P* = 0.0047; Patient-2: Primary vs. Relapsed: *P* = 0.0049; Patient-3: Primary vs. Relapsed: *P* = 0.0016; Patient-4: Primary vs. Relapsed: *P* = 0.0102; Patient-5: Primary vs. Relapsed: *P* = 0.0044; Patient-6: Primary vs. Relapsed: *P* = 0.0018; Patient-7: Primary vs. Relapsed: *P* = 0.0009; Patient-8: Primary vs. Relapsed: *P* = 0.0007; Patient-9: Primary vs. Relapsed: *P* = 0.0030;	3
	E	MCF7: Ri-Vector vs. SALL2-Ri#1: *P* < 0.0001; Ri-Vector vs. SALL2-Ri#2: *P* < 0.0001<br>T47D: Ri-Vector vs. SALL2-Ri#1: *P* < 0.0001; Ri-Vector vs. SALL2-Ri#2: *P* < 0.0001<br>ZR-75-1: Ri-Vector vs. SALL2-Ri#1: *P* < 0.0001; Ri-Vector vs. SALL2-Ri#2: *P* < 0.0001	3
	F	MCF7: Ri-Vector vs. NKX3-1-Ri#1: *P* = 0.7638; Ri-Vector vs. NKX3-1-Ri#2: *P* = 0.0662<br>T47D: Ri-Vector vs. NKX3-1-Ri#1: *P* = 0.0684; Ri-Vector vs. NKX3-1-Ri#2: *P* = 0.5144<br>ZR-75-1: Ri-Vector vs. NKX3-1-Ri#1: *P* = 0.9723; Ri-Vector vs. NKX3-1-Ri#2: *P* = 0.0675	3
EV2	A	MCF7: Vehicle vs. TAM (0.1μM): *P* = 0.1375; Vehicle vs. TAM (1μM): *P* = 0.0004; Vehicle vs. TAM (10μM): *P* < 0.0001	3

		MCF7-TMR: Vehicle vs. TAM (0.1μM): *P* = 0.8533; Vehicle vs. TAM (1μM): *P* = 0.2657; Vehicle vs. TAM (10μM): *P* = 0.1843	
	B	SALL2: MCF7 vs. MCF7-TMR: *P* = 0.0002; ESR1: MCF7 vs. MCF7-TMR: *P* = 0.0005	3
EV3	A	MCF7: Ri-Vector vs. SALL2-Ri#1: *P* < 0.0001; Ri-Vector vs. SALL2-Ri#2: *P* < 0.0001<br>MCF7-TMR: Vector vs. SALL2: *P* < 0.0001; ZR-75-30: Vector vs. SALL2: *P* < 0.0001	6
	B	MCF7: Ri-Vector: TAM- vs. TAM+: *P* = 0.0025; SALL2-Ri#1: TAM- vs. TAM+: *P* = 0.4798; SALL2-Ri#2: TAM- vs. TAM+: *P* = 0.8640<br>MCF7-TMR: Vector: TAM- vs. TAM+: *P* = 0.4828; SALL2: TAM- vs. TAM+: *P* = 0.0385<br>ZR-75-30: Vector: TAM- vs. TAM+: *P* = 0.8908; SALL2: TAM- vs. TAM+: *P* = 0.0254	3
	C	MCF7/Vehicle: Ri-Vector vs. SALL2-Ri#1: *P* = 0.0135; SALL2-Ri#1 vs.SALL2-Ri#1+ESR1: *P* = 0.5122<br>MCF7/E2: Ri-Vector vs. SALL2-Ri#1: *P* < 0.0001; SALL2-Ri#1 vs.SALL2-Ri#1+ESR1: *P* = 0.0016<br>MCF7/TAM: Ri-Vector vs. SALL2-Ri#1: *P* = 0.0004; SALL2-Ri#1 vs.SALL2-Ri#1+ESR1: *P* = 0.0013<br>MCF7-TMR/Vehicle: Vector vs. SALL2: *P* = 0.0080; SALL2 vs. SALL2+ESR1-Ri: *P* = 0.8589<br>MCF7-TMR/E2: Vector vs. SALL2: *P* < 0.0001; SALL2 vs. SALL2+ESR1-Ri: *P* < 0.0001<br>MCF7-TMR/TAM: Vector vs. SALL2: *P* = 0.0038; SALL2 vs. SALL2+ESR1-Ri: *P* = 0.0134<br>ZR-75-30/Vehicle: Vector vs. SALL2: *P* = 0.0027; SALL2 vs. SALL2+ESR1-Ri: *P* = 0.8819<br>ZR-75-30/E2: Vector vs. SALL2: *P* = 0.0005; SALL2 vs. SALL2+ESR1-Ri: *P* < 0.0001<br>ZR-75-30/TAM: Vector vs. SALL2: *P* = 0.0011; SALL2 vs. SALL2+ESR1-Ri: *P* = 0.0017	3
	D	MCF7/Vehicle: Ri-Vector vs. SALL2-Ri#1: *P* = 0.0330; SALL2-Ri#1 vs.SALL2-Ri#1+ESR1: *P* = 0.9006<br>MCF7/TAM: Ri-Vector vs. SALL2-Ri#1: *P* < 0.0001;	3

		SALL2-Ri#1 vs.SALL2-Ri#1+ESR1: *P* = 0.0003<br>MCF7-TMR/Vehicle: Vector vs. SALL2: *P* = 0.0022; SALL2 vs. SALL2+ESR1-Ri: *P* = 0.9331<br>MCF7-TMR/TAM: Vector vs. SALL2: *P* < 0.0001; SALL2 vs. SALL2+ESR1-Ri: *P* < 0.0001<br>ZR-75-30/Vehicle: Vector vs. SALL2: *P* = 0.0075; SALL2 vs. SALL2+ESR1-Ri: *P* = 0.8658<br>ZR-75-30/TAM: Vector vs. SALL2: *P* < 0.0001; SALL2 vs. SALL2+ESR1-Ri: *P* < 0.0001	
EV4	C	BT-20: Vector vs. SALL2: *P* = 0.0017; BT-549: Vector vs. SALL2: *P* = 0.7717; MDA-MB-231: Vector vs. SALL2: *P* = 0.2593	3
	D	BT-20: Vector vs. SALL2: *P* < 0.0001; BT-549: Vector vs. SALL2: *P* = 0.9868; MDA-MB-231: Vector vs. SALL2: *P* = 0.6365	6
EV5	A	Vehicle vs. 5-Aza-dC: *P* = 0.8563; Vehicle vs. TAM: *P* = 0.3804; Vehicle vs. 5-Aza-dC+TAM: *P* = 0.3461	3
	B	Vehicle vs. 5-Aza-dC: *P* = 0.2083; Vehicle vs. TAM: *P* = 0.7847; Vehicle vs. 5-Aza-dC+TAM: *P* = 0.9991	3
	D	TAM-: 5-Aza-dC- vs. 5-Aza-dC+: *P* = 0.8148; TAM+: 5-Aza-dC- vs. 5-Aza-dC+: *P* = 0.2511	8
	E	Ki67: Vehicle vs. 5-Aza-dC: *P* = 0.7072; Vehicle vs. TAM: *P* = 0.3894; Vehicle vs. 5-Aza-dC+TAM: *P* = 0.8039<br>Apoptosis: Vehicle vs. 5-Aza-dC: *P* = 0.0745; Vehicle vs. TAM: *P* = 0.5765; Vehicle vs. 5-Aza-dC+TAM: *P* = 0.0549	8
S3	B	Normal vs. Primary: *P* = 0.0018; Normal vs. Relapsed: *P* = 0.0370; Primary vs. Relapsed : *P* < 0.0001	Normal: n = 12; Primary: n = 90; Relapsed: n = 21
	C	ER+ vs. ER-: *P* = 0.0306	3
S4	B	MCF7: Ri-Vector vs. SALL2-Ri#1: *P* < 0.0001; Ri-Vector vs. SALL2-Ri#2: *P* < 0.0001<br>MCF7-TMR: Vector vs. SALL2: *P* = 0.0008; ZR-75-30: Vector vs. SALL2: *P* = 0.0011	3
	C	Vct: Ri-Vector vs. SALL2-Ri#1: *P* = 0.2808; Ri-Vector vs. SALL2-Ri#2: *P* = 0.4437	3

	Wt: Ri-Vector vs. SALL2-Ri#1: *P* = 0.0015; Ri-Vector vs. SALL2-Ri#2: *P* = 0.0009<br>Mut1: Ri-Vector vs. SALL2-Ri#1: *P* = 0.2529; Ri-Vector vs. SALL2-Ri#2: *P* = 0.1829<br>Mut2: Ri-Vector vs. SALL2-Ri#1: *P* = 0.0025; Ri-Vector vs. SALL2-Ri#2: *P* = 0.0021	
D	P1-SALL2: Ri-Vector vs. SALL2-Ri#1: *P* = 0.1000; Ri-Vector vs. SALL2-Ri#2: *P* = 0.4315<br>P1-IgG: Ri-Vector vs. SALL2-Ri#1: *P* < 0.0001; Ri-Vector vs. SALL2-Ri#2: *P* < 0.0001<br>P2-SALL2: Ri-Vector vs. SALL2-Ri#1: *P* = 0.1861; Ri-Vector vs. SALL2-Ri#2: *P* = 0.2749<br>P2-IgG: Ri-Vector vs. SALL2-Ri#1: *P* = 0.0519; Ri-Vector vs. SALL2-Ri#2: *P* = 0.0893	3
E	p300: Ri-Vector vs. SALL2-Ri#1: *P* < 0.0001; Ri-Vector vs. SALL2-Ri#2: *P* < 0.0001<br>RNAPII: Ri-Vector vs. SALL2-Ri#1: *P* < 0.0001; Ri-Vector vs. SALL2-Ri#2: *P* < 0.0001<br>H3K4me3: Ri-Vector vs. SALL2-Ri#1: *P* < 0.0001; Ri-Vector vs. SALL2-Ri#2: *P* = 0.0002	3
