# Supplementary material for: Epigenetic silencing of SALL2 confers tamoxifen resistance in breast cancer
Source: EMBO Mol Med. 2019 Oct 28;11(12):e10638. doi: 10.15252/emmm.201910638 (PMC6895605; doi:10.15252/emmm.201910638)
Supplement: Supplementary file 2 — Expanded View Figures PDF [file EMMM-11-e10638-s002.pdf]

Expanded View Figures

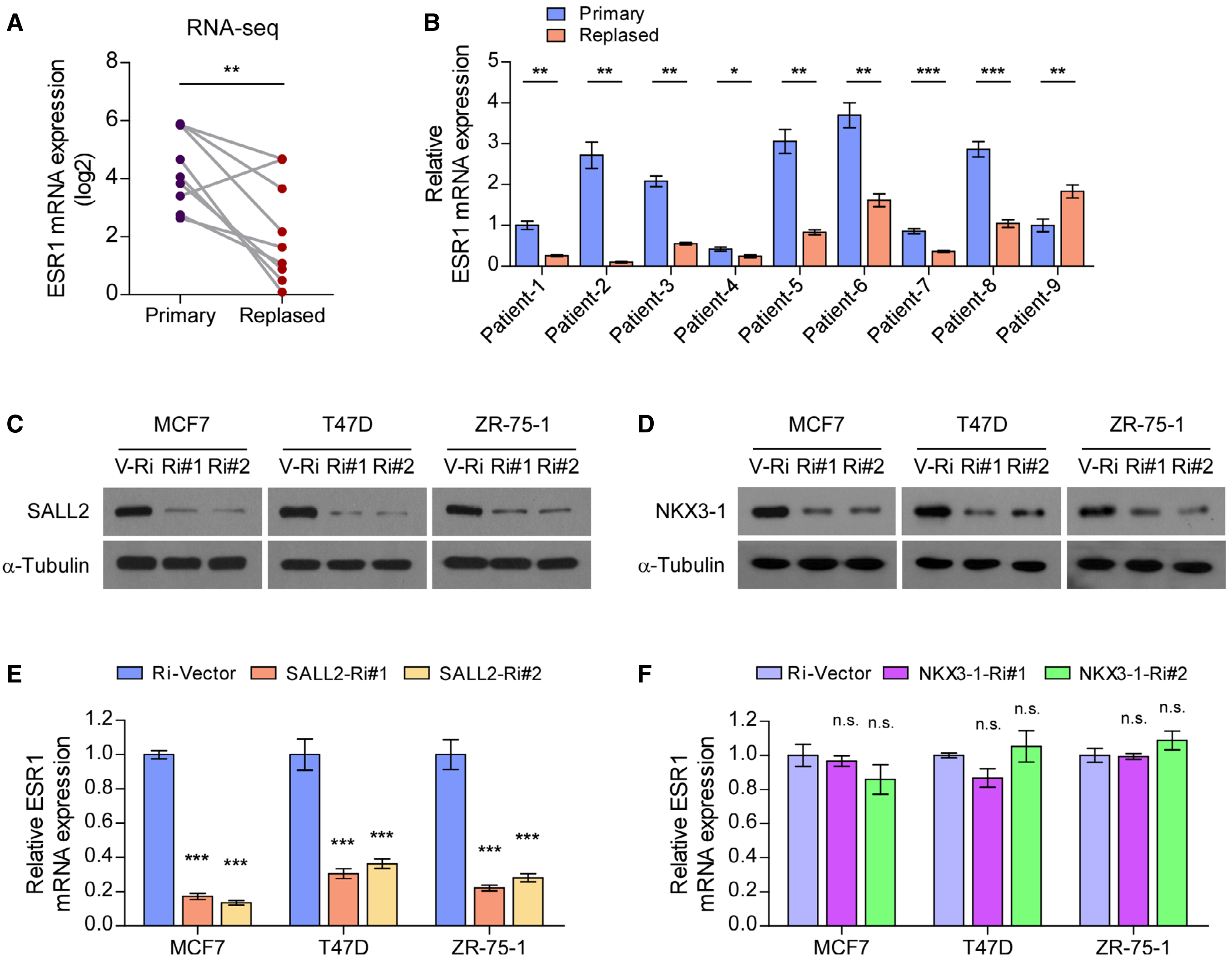

Figure EV1. SALL2 transcriptionally upregulates ESR1 in breast cancer.

A RNA-seq analysis of ESR1 mRNA levels in 9 paired pre-tamoxifen-treated primary breast cancer tissues and relapsed tamoxifen-resistant breast cancer tissues.

B qRT–PCR analysis of *ESR1* expression in 9 paired pre-tamoxifen-treated primary breast cancer tissues and relapsed tamoxifen-resistant breast cancer tissues. *GAPDH* was used as an internal control.

C, D WB analysis of SALL2 (A) and NKX3-1 (B) expression in the indicated cells transfected with Ri-Vector (V-Ri) or shRNAs (Ri#1/2) against SALL2 or NKX3-1. α-Tubulin was used as the loading control.

E, F qRT–PCR analysis of *ESR1* expression in the indicated cells transfected with Ri-Vector or shRNAs (Ri#1/2) against SALL2 or NKX3-1.

Data information: In (A), *P*-values were determined by two-tailed paired Student's *t*-test. In (B), data are presented as mean ± SD, and *P*-values were determined by two-tailed unpaired Student's *t*-test, $n = 3$. In (E and F), data are presented as mean ± SD, and *P*-values were determined by one-way ANOVA test, $n = 3$. *$P < 0.05$, **$P < 0.01$, ***$P < 0.001$, n.s., no significance. Exact *P*-values are specified in Appendix Table S10.
Source data are available online for this figure.

© 2019 The Authors

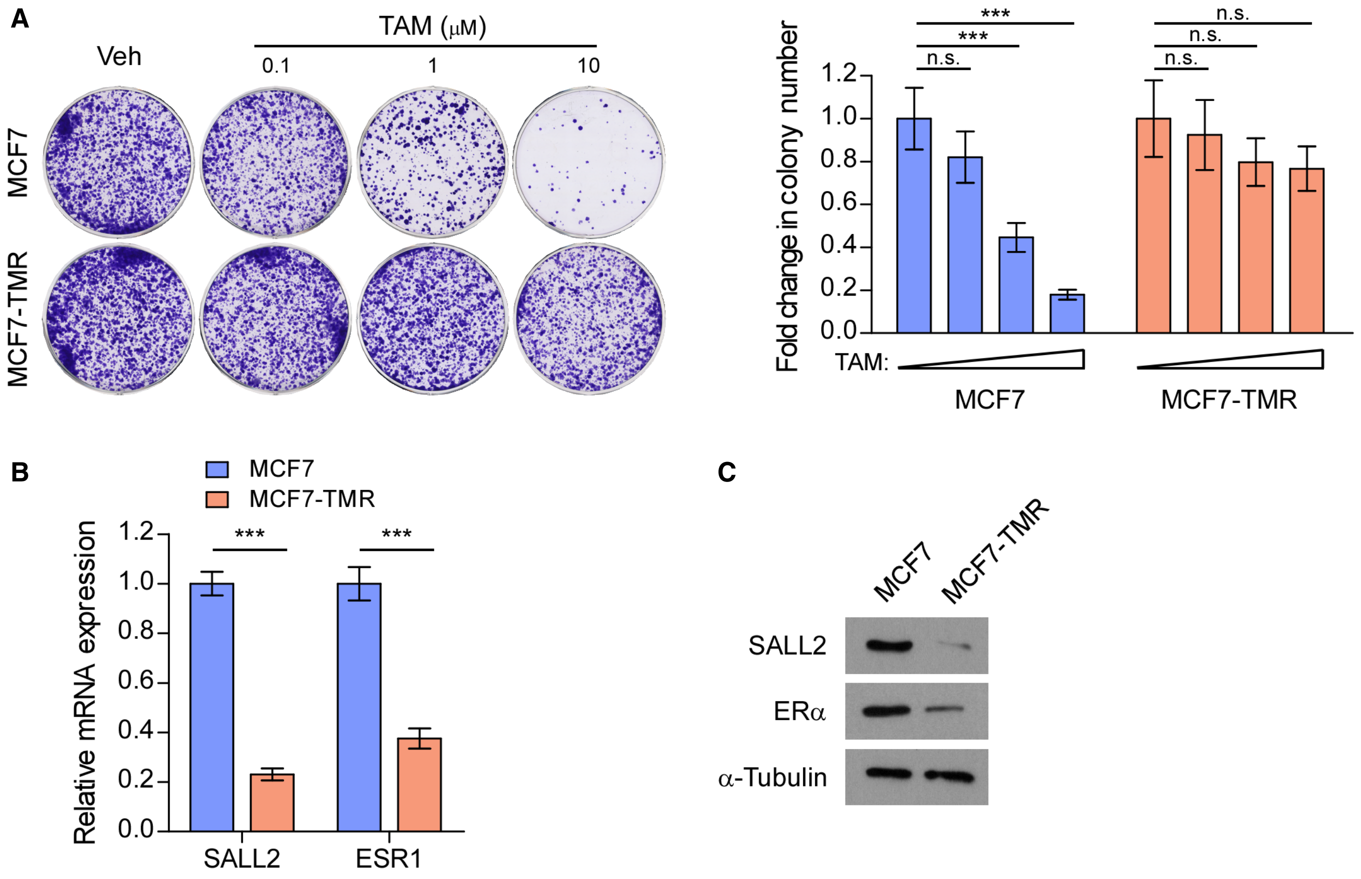

Figure EV2. Establishing MCF7/tamoxifen-resistant (MCF7-TMR) cell line.

A Representative images (left panel) and quantification (right panel) of colony formation by MCF7 and MCF7-TMR cell lines with increasing doses of tamoxifen (TAM) treatment.
B qRT–PCR analysis of *SALL2* and *ESR1* expression in MCF7 and MCF7-TMR cell lines. *GAPDH* was used as an internal control.
C WB analysis of SALL2 and ERα expression in MCF7 and MCF7-TMR cell lines. α-Tubulin was used as a loading control.

Data information: In (A), data are presented as mean ± SD, and *P*-values were determined by one-way ANOVA test, $n = 3$. In (B), data are presented as mean ± SD, and *P*-values were determined by two-tailed Student's *t*-test, $n = 3$. ***$P < 0.001$, n.s., no significance. Exact *P*-values are specified in Appendix Table S10.
Source data are available online for this figure.

Figure EV3. Silencing SALL2 promotes tamoxifen resistance via downregulation of ESR1.

A Cell viability was assessed in the indicated cells treated with increasing doses of TAM for 5 days.
B FACS analysis of cell cycle of the indicated breast cancer cell lines treated with or without TAM (1 µM).
C Quantification of crystal violet-stained colony formed by the indicated cells treated with E2 (10 nM) or TAM (1 µM).
D Annexin V–FITC/PI staining of the indicated TAM (10 µM)- or vehicle (Veh)-treated cells.

Data information: In (A, C, and D), data are presented as mean ± SD, and *P*-values were determined by two-way ANOVA test, $n = 6$ in (A), $n = 3$ in (C and D). In (B), *P*-values were determined by χ^2 test, $n = 3$. *$P < 0.05$, **$P < 0.01$, ***$P < 0.001$, n.s., no significance. Exact *P*-values are specified in Appendix Table S10.

© 2019 The Authors

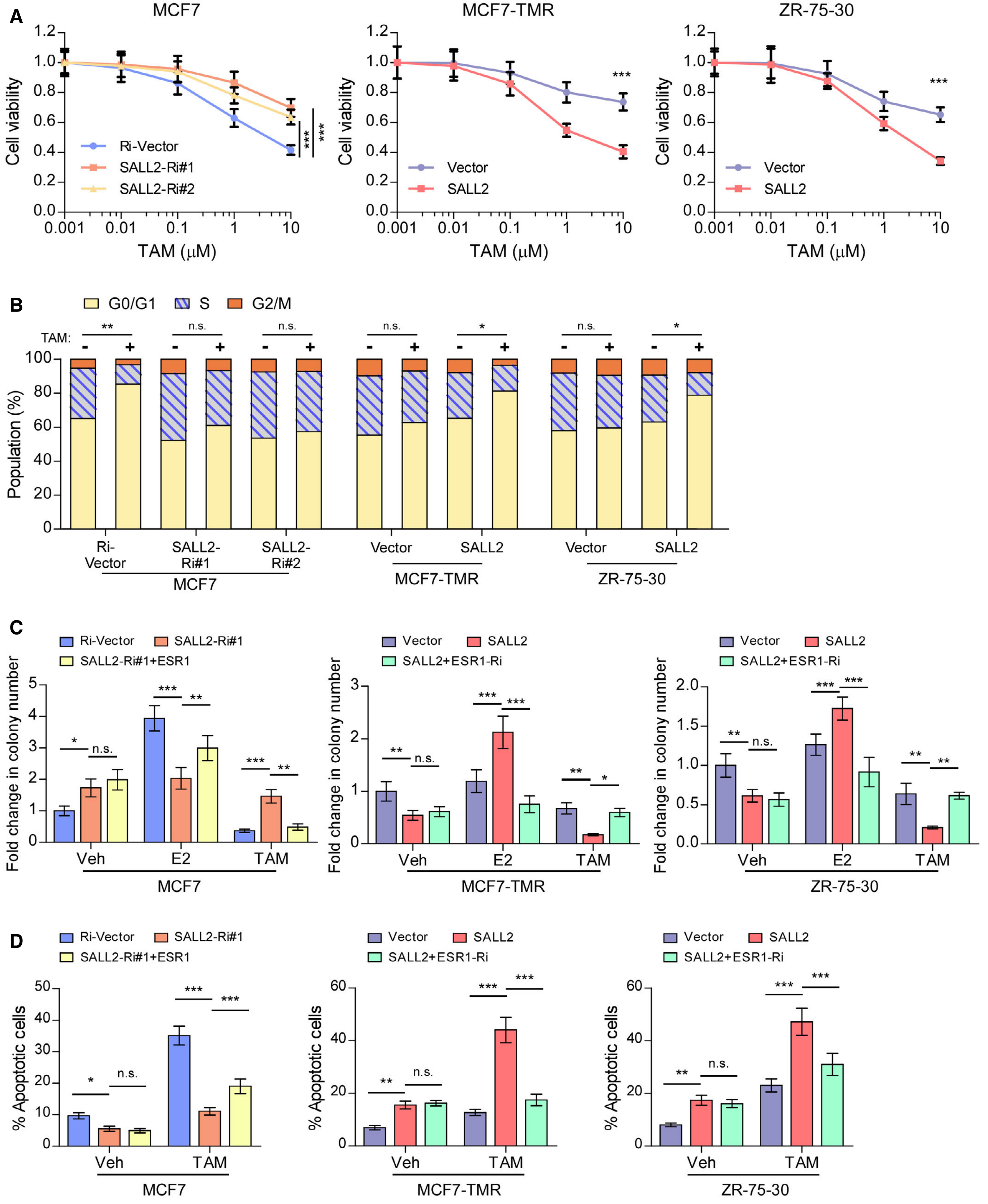

Figure EV3.

 © 2019 The Authors

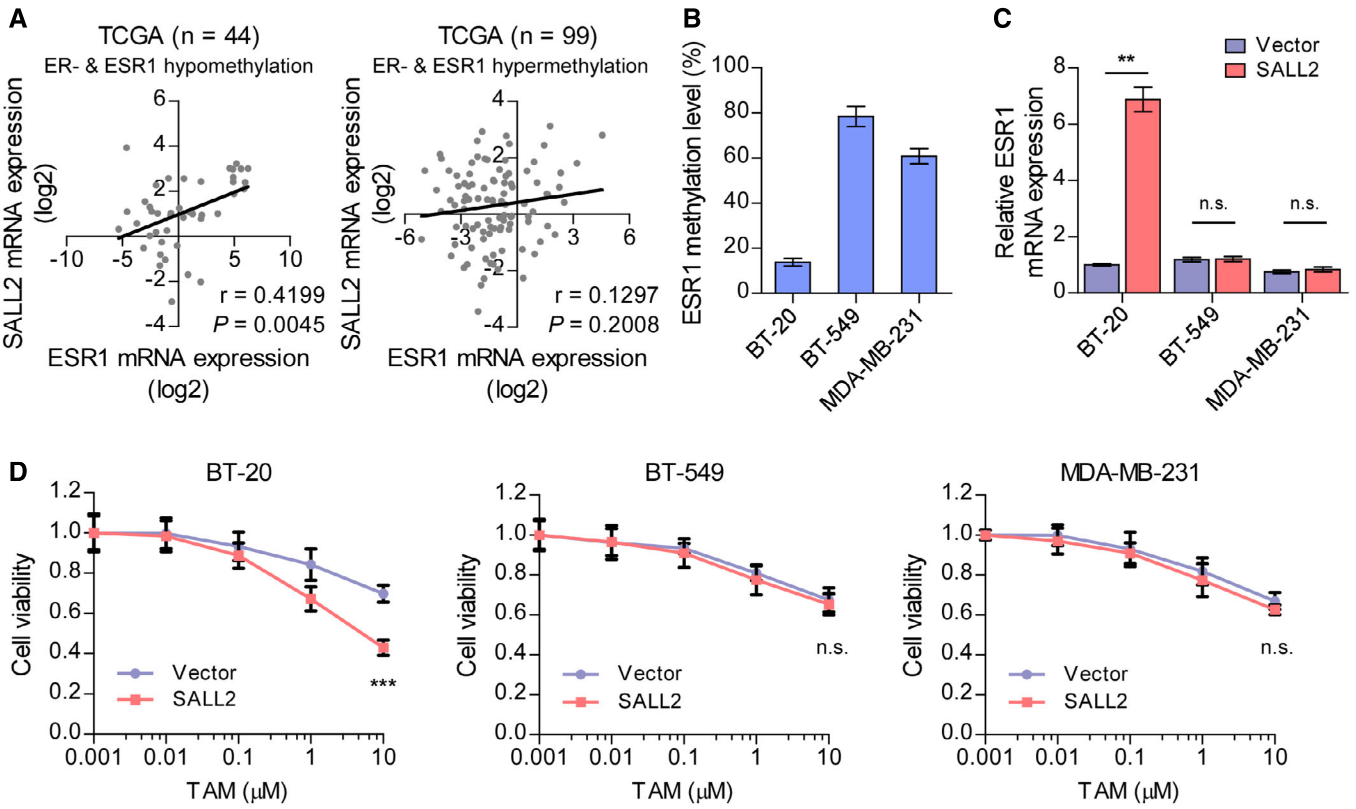

Figure EV4. SALL2 is decreased in ER- breast cancer cell lines and tissues.

A Scatter diagram and linear regression analysis of correlation between ESR1 and SALL2 levels in ESR1-hypomethylated (β value < 0.6, left panel) and ESR1-hypermethylated (β value $\geq$ 0.6, right panel) ER^- breast cancer patients in the TCGA (BRCA) dataset.

B BSP analysis of methylation status at ESR1 promoter in the indicated ER^- breast cancer cell lines.

C qRT–PCR analysis of *ESR1* expression in the indicated cells. *GAPDH* was used as an internal control in qRT–PCR analysis.

D Cell viability was examined in the indicated cells treated with increasing doses of TAM for 5 days.

Data information: In (A), r-values were determined by Pearson's test, and *P*-values were determined by two-tailed Student's *t*-test. In (B), data are presented as mean ± SD, $n = 3$. In (C), data are presented as mean ± SD, and *P*-values were determined by two-tailed Student's *t*-test, $n = 3$. In (D), *P*-values were determined by two-way ANOVA test, $n = 6$. $^{**}P < 0.01$, $^{***}P < 0.001$, n.s., no significance. Exact *P*-values are specified in Appendix Table S10.

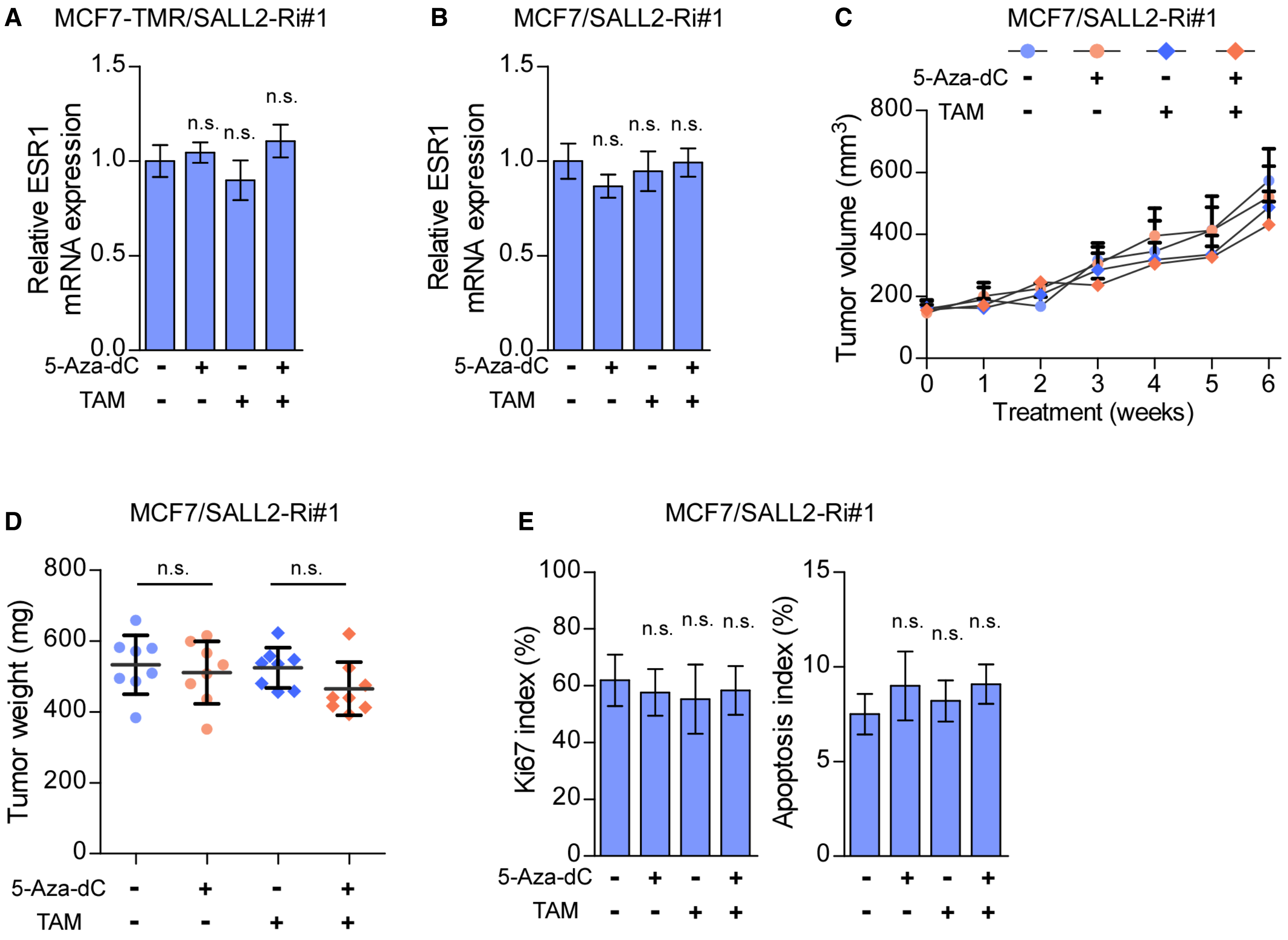

Figure EV5. Overexpression of SALL2 restores sensitivity of resistant breast cancer cells to tamoxifen.

A, B qRT–PCR analysis of *ESR1* expression in the MCF7-TMR/SALL2-Ri#1 cells (A) and MCF7/SALL2-Ri#1 cells (B) with the indicated treatments. *GAPDH* was used as an internal control.

C Tumor growth curves of the indicated MCF7/SALL2-Ri#1/xenograft tumors (n = 8/group) upon 5-Aza-dC and TAM treatment. 5-Aza-dC treatment was started when the tumors reached approximately 200 mm^3.

D Quantification of xenograft tumor weight at the end of the experiment shown in (C) (n = 8/group).

E The proliferation index was determined using the percentage of Ki67-positive cells, and the apoptosis index was determined using the percentage of TUNEL-positive cells, in the MCF7/SALL2-Ri xenograft tumors upon 5-Aza-dC and TAM treatment (n = 8/group).

Data information: In (A, B, D, and E), data are presented as mean ± SD, and P-values were determined by one-way ANOVA test, n = 3 in (A and B). In (C), data are presented as mean ± SD, and P-values were determined by two-way ANOVA test. n.s., no significance. Exact P-values are specified in Appendix Table S10.

© 2019 The Authors
