# Supplementary figures and images for: Epigenetic silencing of SALL2 confers tamoxifen resistance in breast cancer
Source: EMBO Mol Med. 2019 Oct 28;11(12):e10638. doi: 10.15252/emmm.201910638 (PMC6895605; doi:10.15252/emmm.201910638)

Figure EV1

C

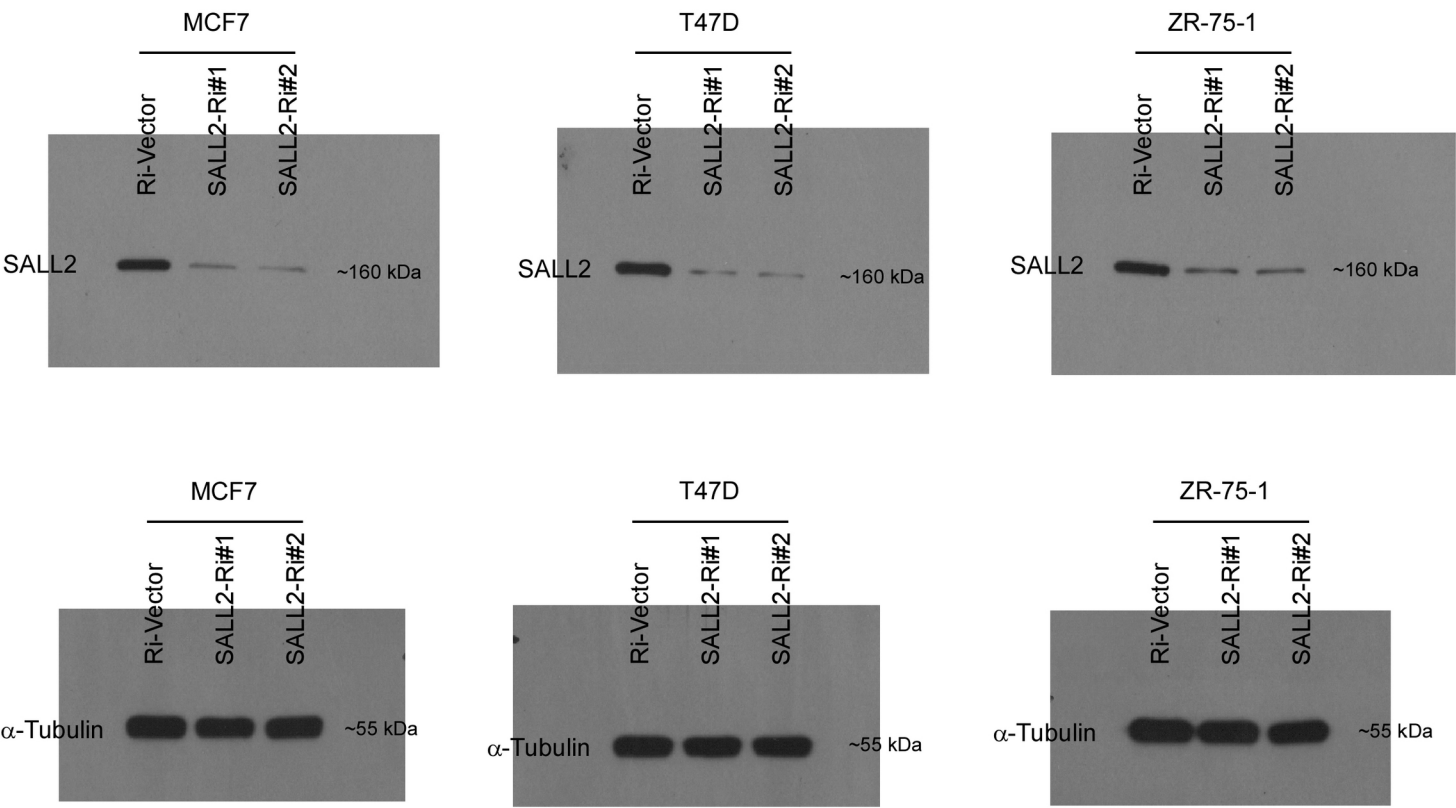

D

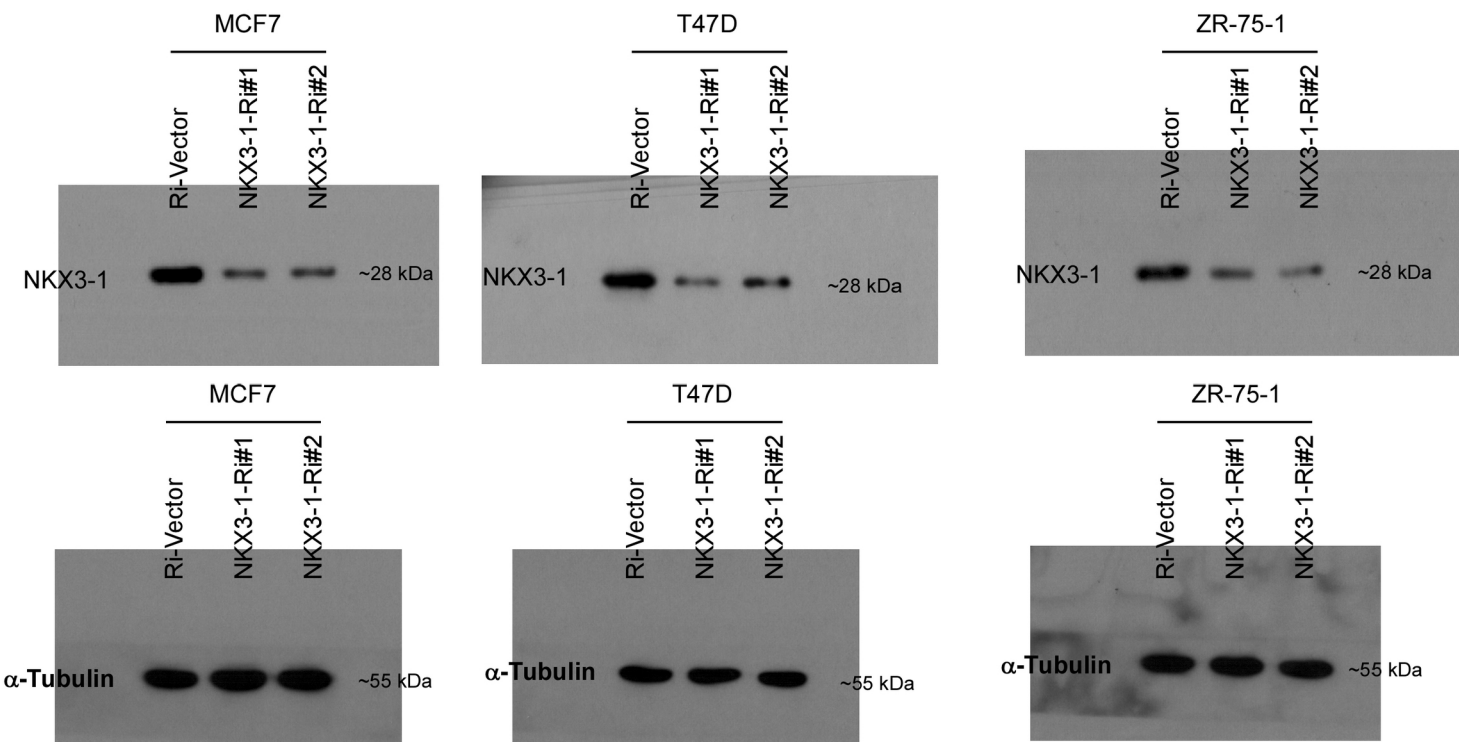

Supplement: Supplementary file 3 — Source Data for Expanded View and Appendix [file EMMM-11-e10638-s008.zip › 10638_EV_appendix_sd/EMM-2019-10638_SourceDataForEVFigure1.pdf]

Figure EV2

C

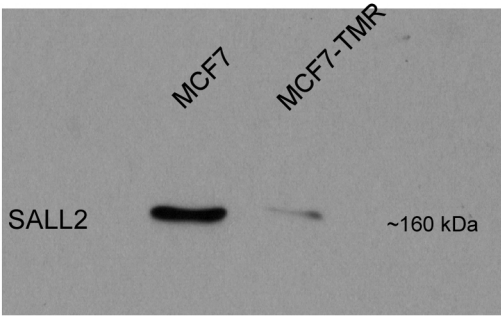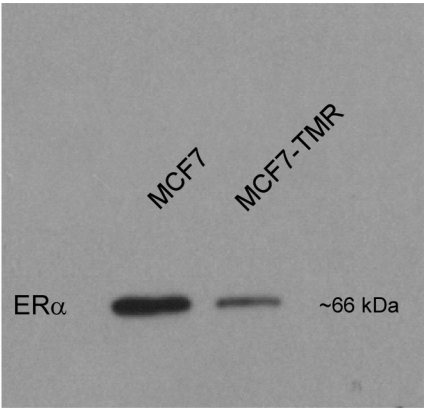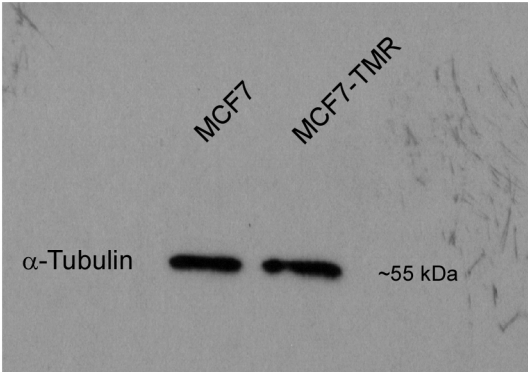

Supplement: Supplementary file 3 — Source Data for Expanded View and Appendix [file EMMM-11-e10638-s008.zip › 10638_EV_appendix_sd/EMM-2019-10638_SourceDataForEVFigure2.pdf]

## F

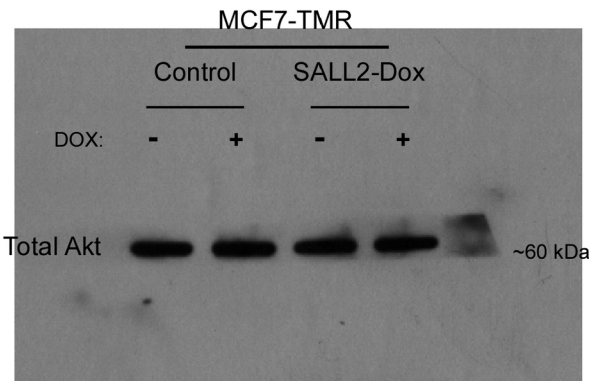

Supplement: Supplementary file 3 — Source Data for Expanded View and Appendix [file EMMM-11-e10638-s008.zip › 10638_EV_appendix_sd/EMM-2019-10638_SourceDataForAppendixFigureS4.pdf]

Figure 3

B

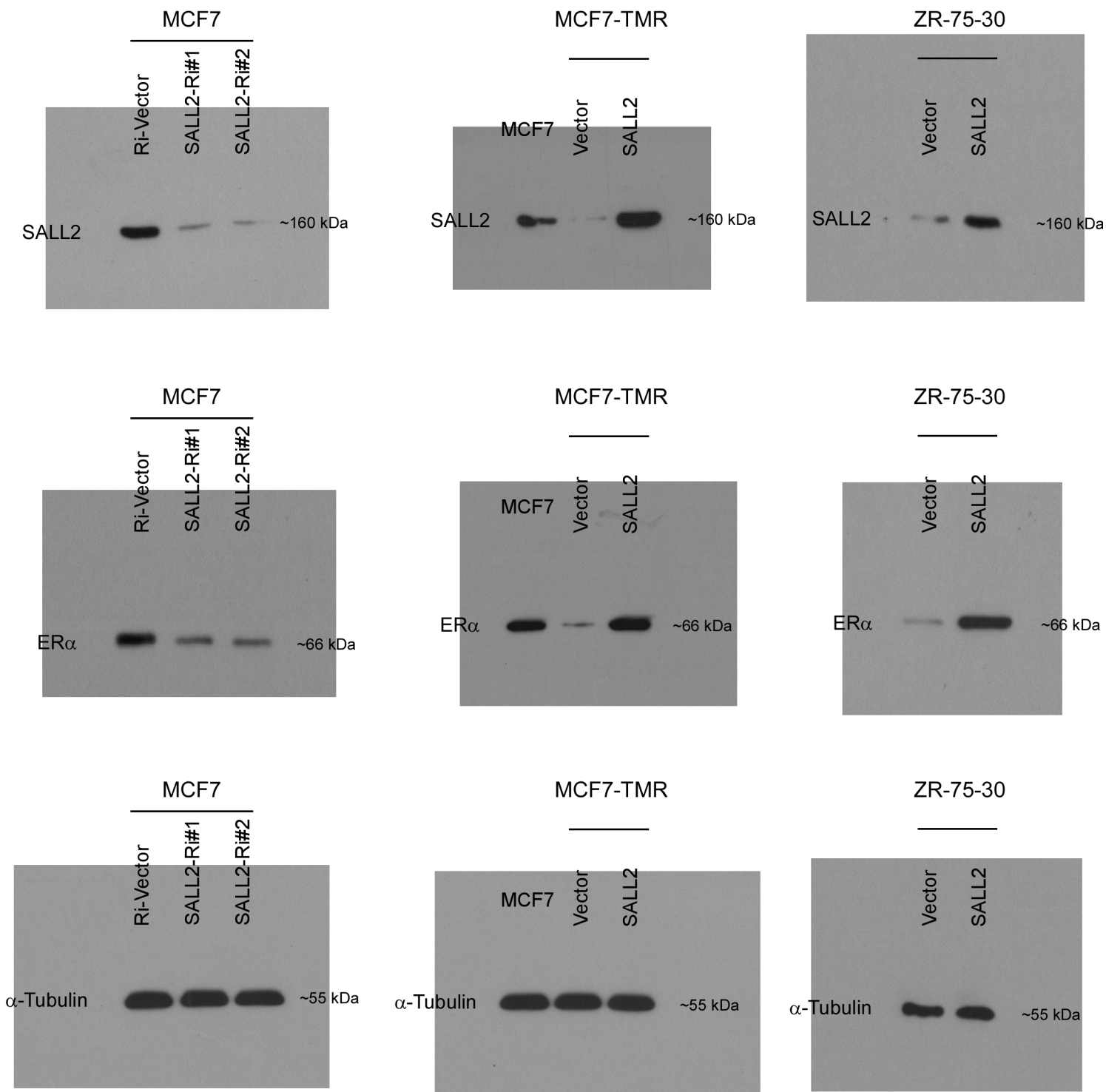

Supplement: Supplementary file 6 — Source Data for Figure 3 [file EMMM-11-e10638-s004.pdf]

Figure 4

F

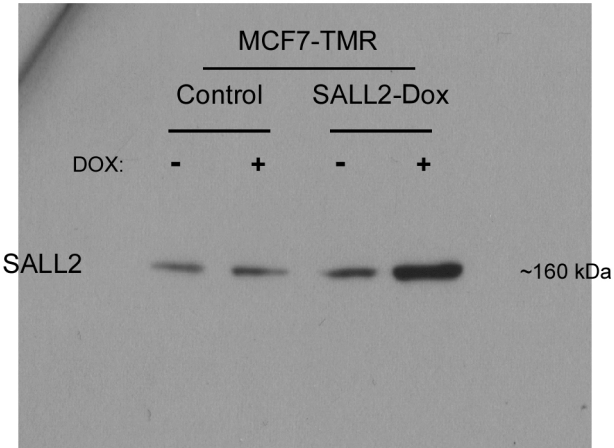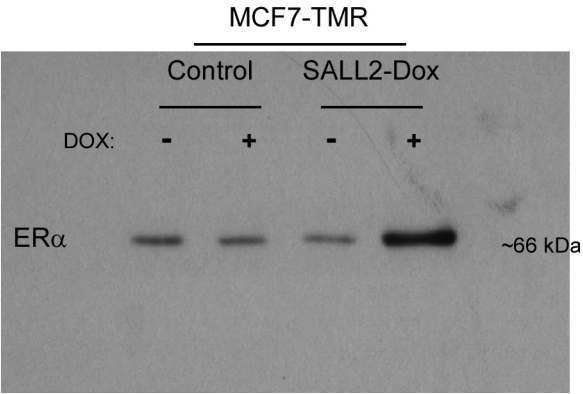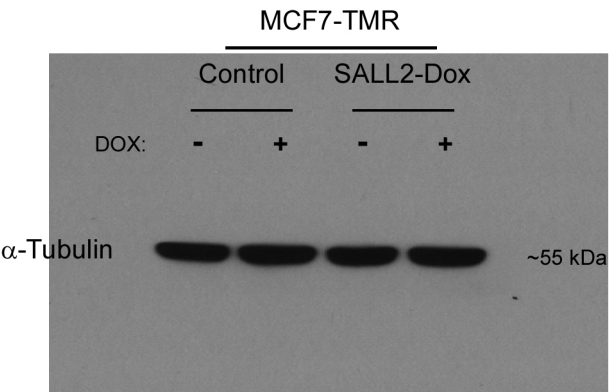

Supplement: Supplementary file 7 — Source Data for Figure 4 [file EMMM-11-e10638-s005.pdf]

Figure 5

B

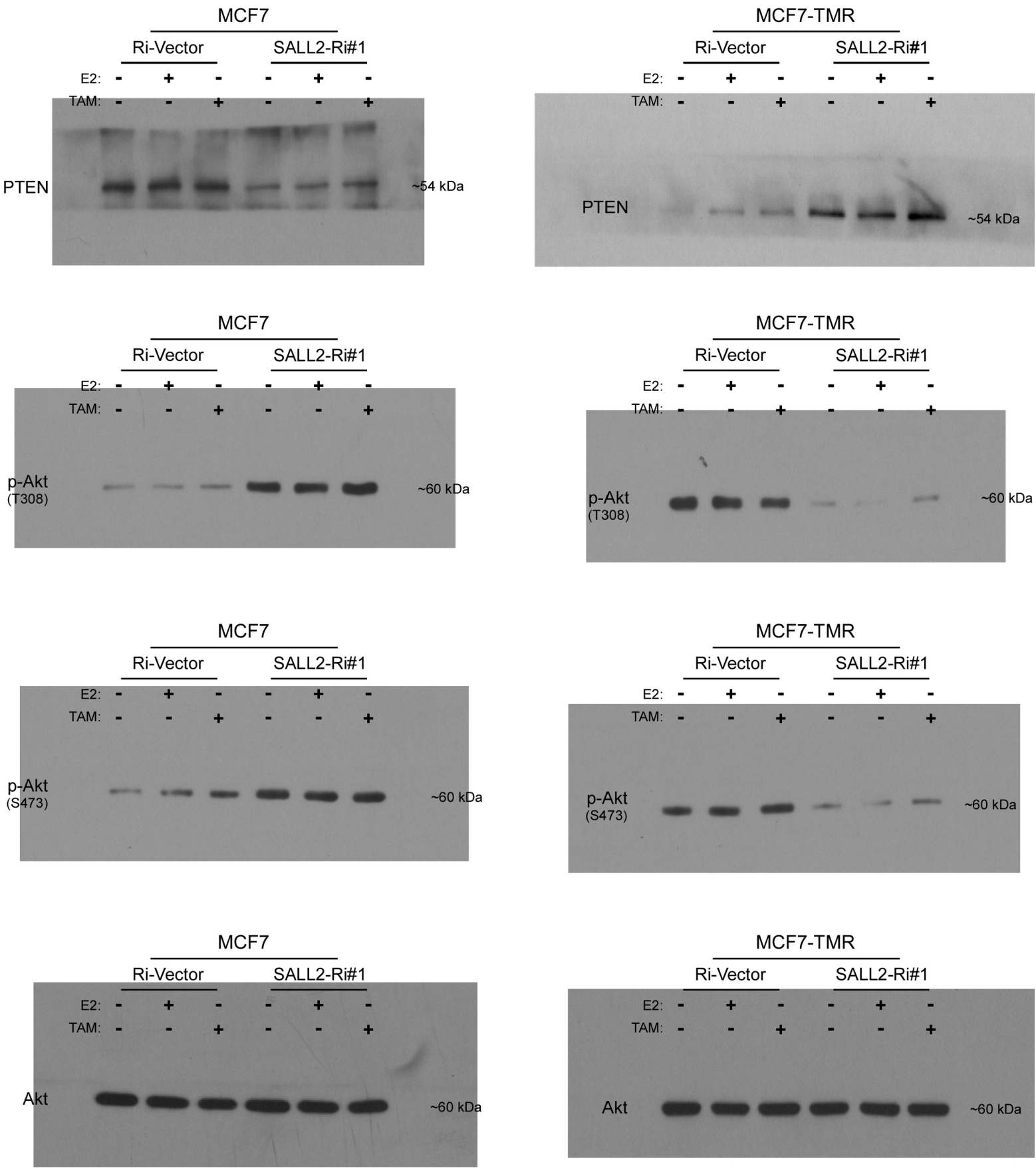

Figure 5

B

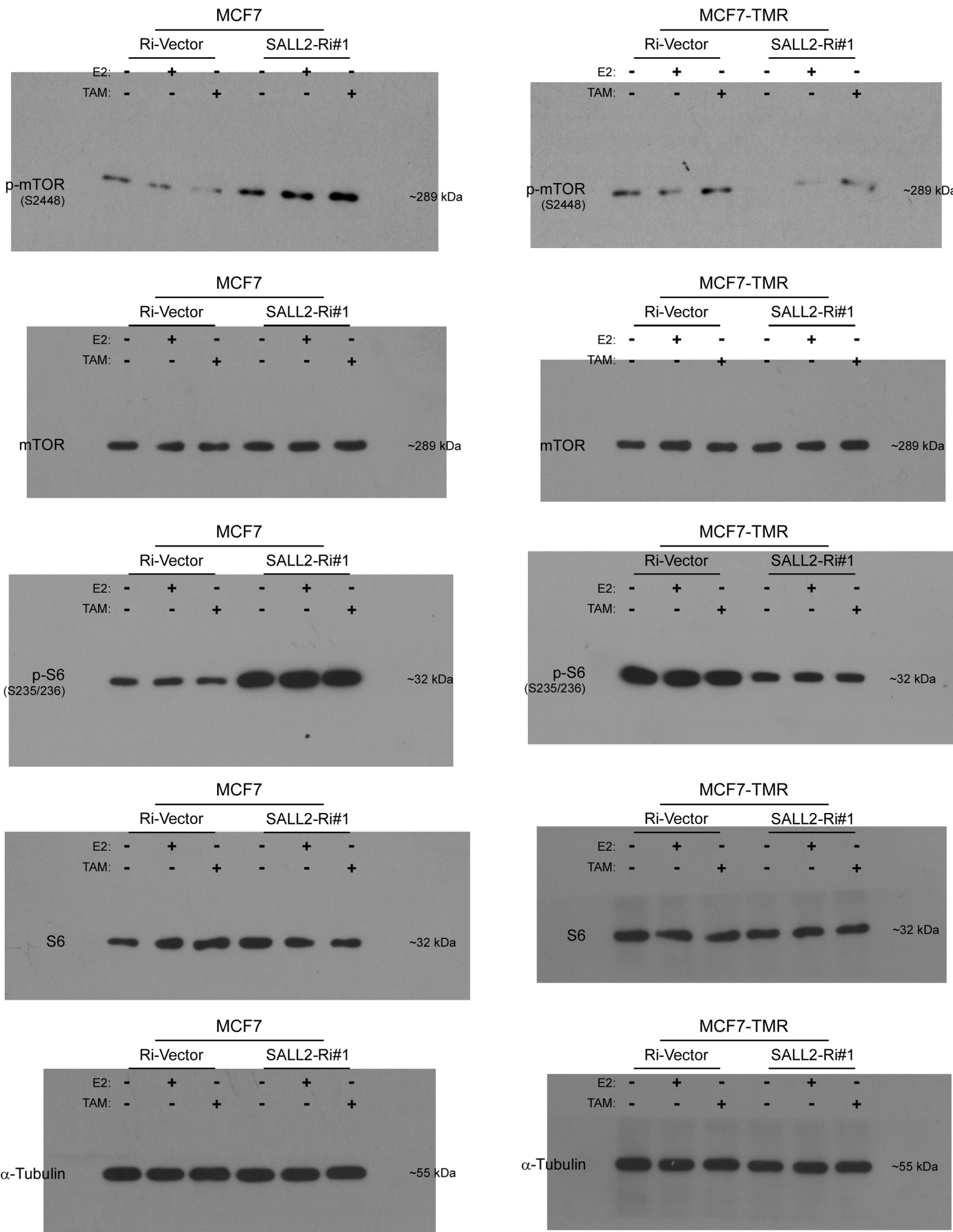

Figure 5

C

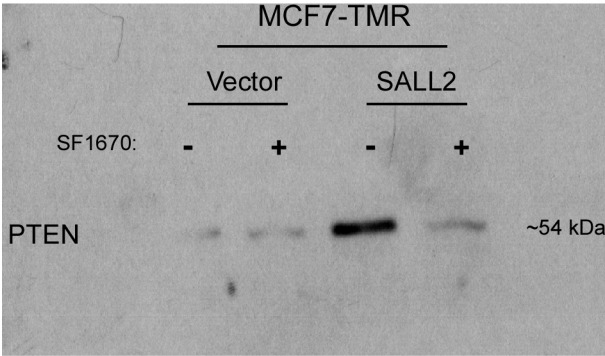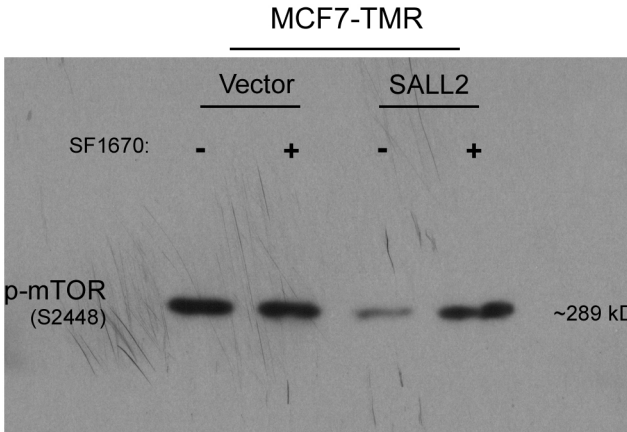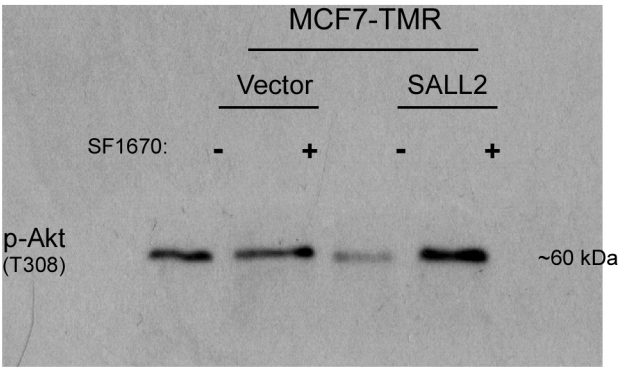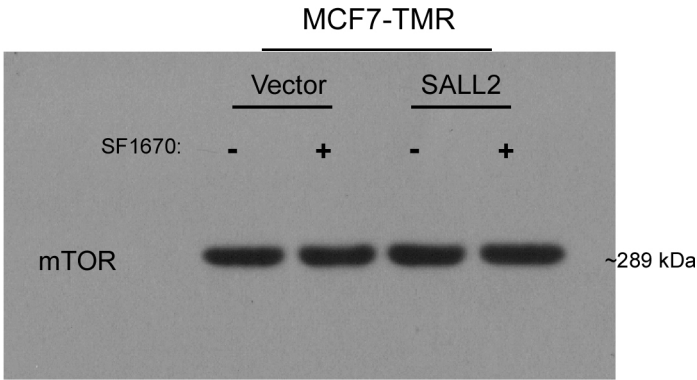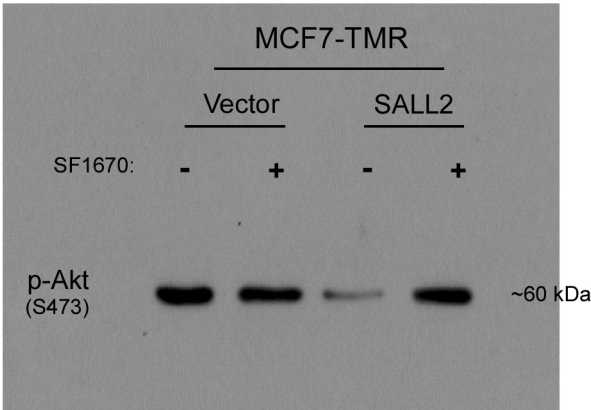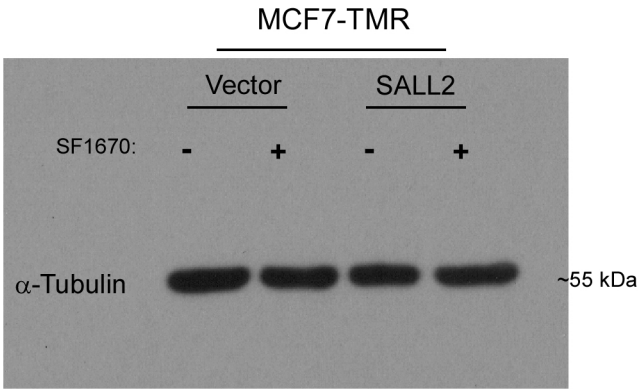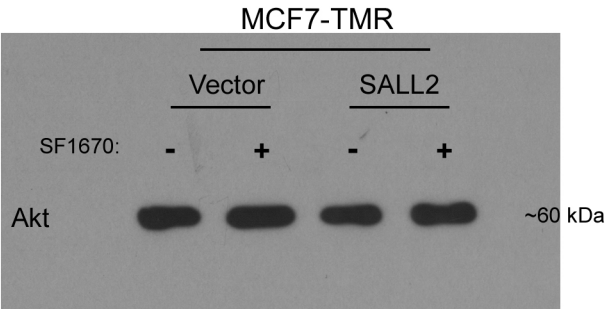

Supplement: Supplementary file 8 — Source Data for Figure 5 [file EMMM-11-e10638-s006.pdf]

Figure 7

E

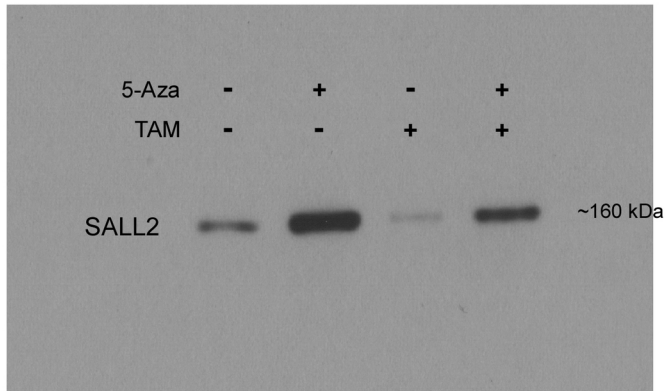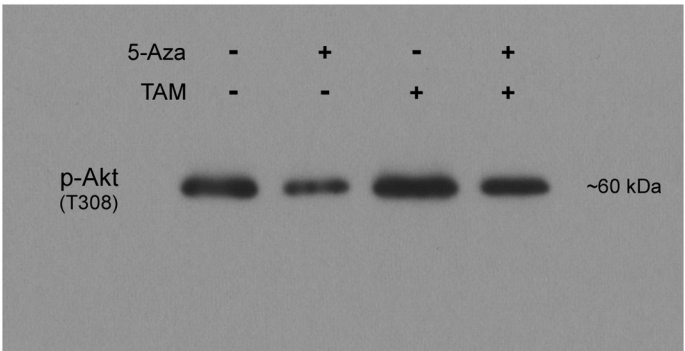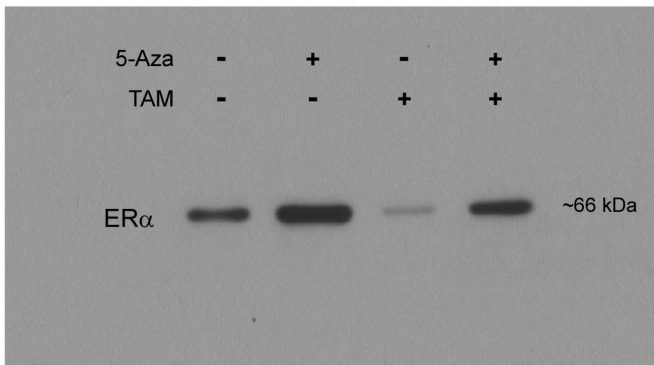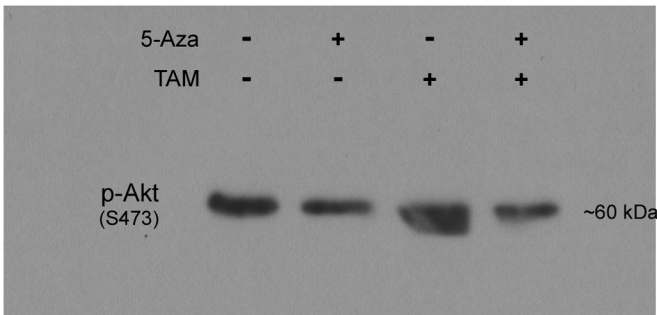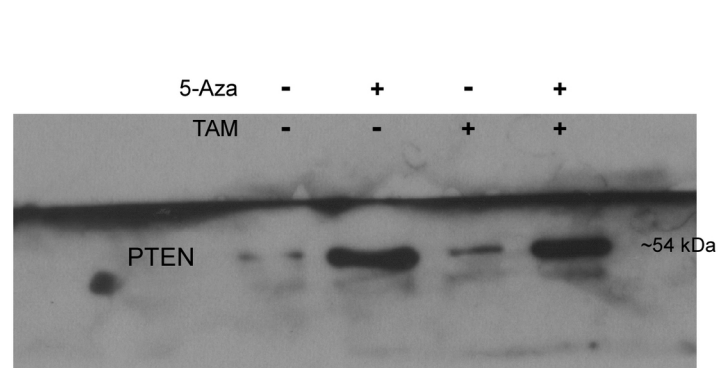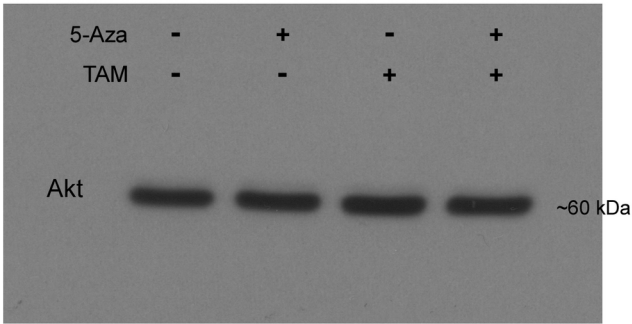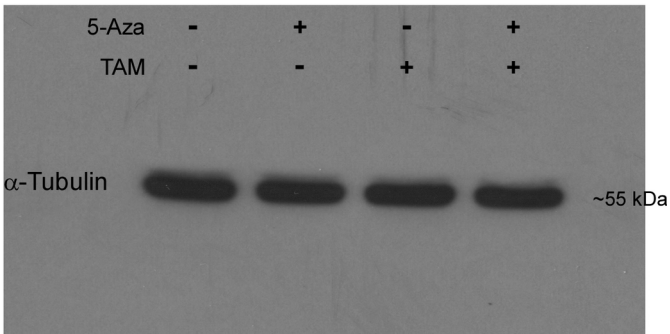

Supplement: Supplementary file 9 — Source Data for Figure 7 [file EMMM-11-e10638-s007.pdf]
