# Supplementary figures and images for: Epigenetic silencing of SALL2 confers tamoxifen resistance in breast cancer
Source: EMBO Mol Med. 2022 Mar 7;14(3):e15618. doi: 10.15252/emmm.202115618 (PMC8899907; doi:10.15252/emmm.202115618)

**Figure 5G**

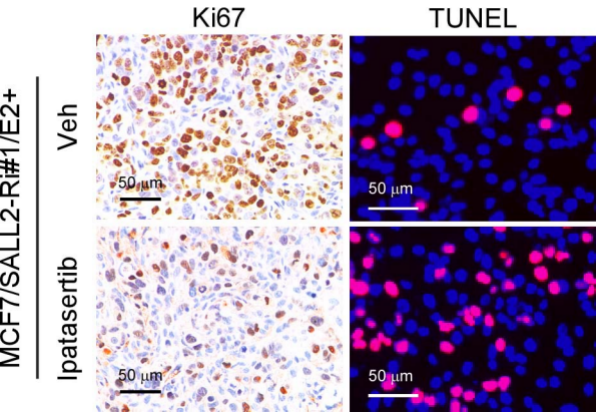

Supplement: Supplementary file 1 — Source Data for Figure 5G [file EMMM-14-e15618-s001.zip › Figure-5G-source-data/Corrected_Figure_5G.pdf]

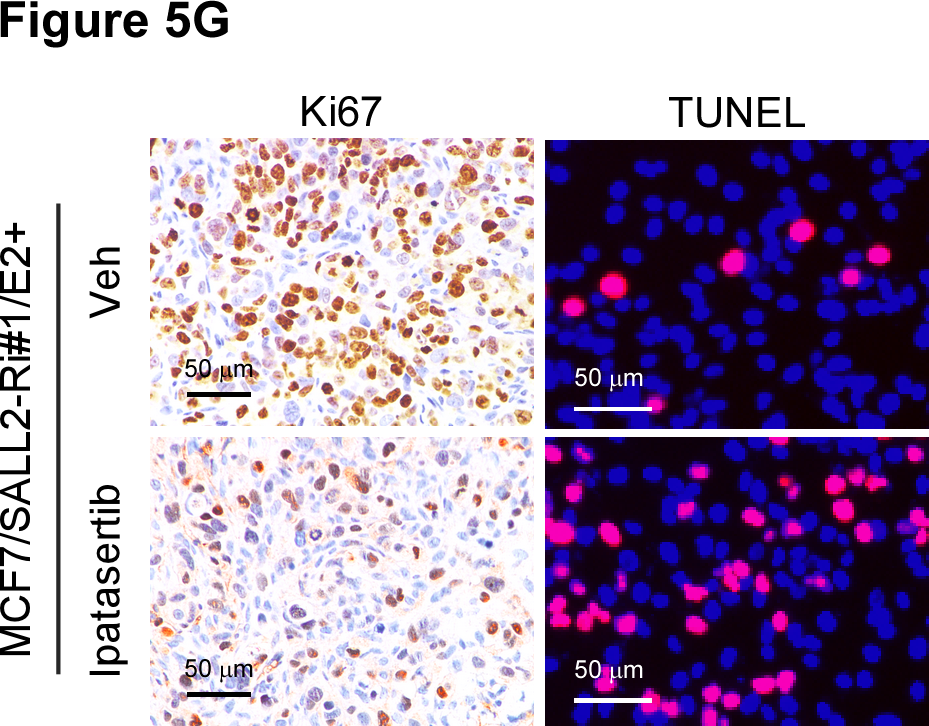

Supplement: Supplementary file 1 — Source Data for Figure 5G [file EMMM-14-e15618-s001.zip › Figure-5G-source-data/Corrected_Figure_5G.tif]

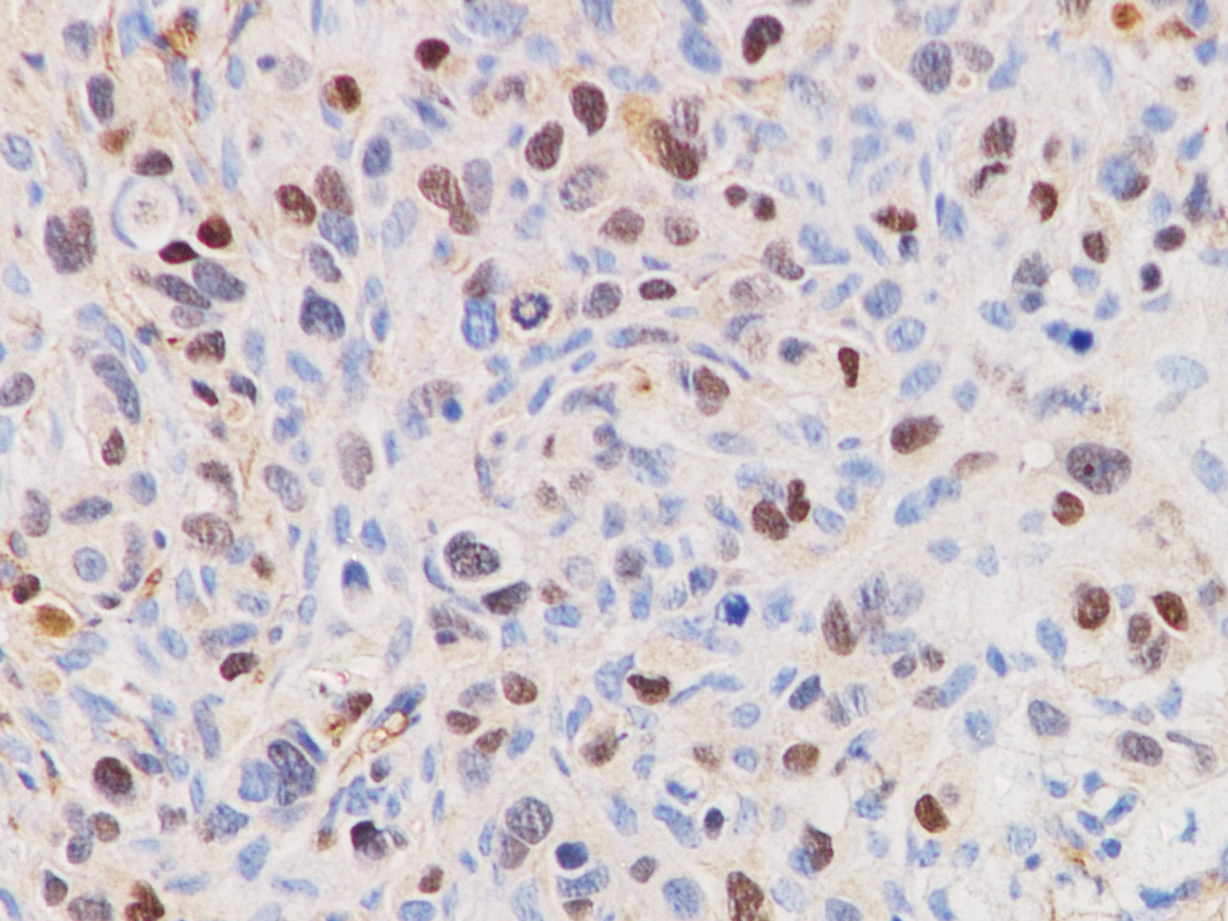

Supplement: Supplementary file 1 — Source Data for Figure 5G [file EMMM-14-e15618-s001.zip › Figure-5G-source-data/MCF7-SALL2-Ri#1-E2+-Ipatasertib-Ki67.tif]

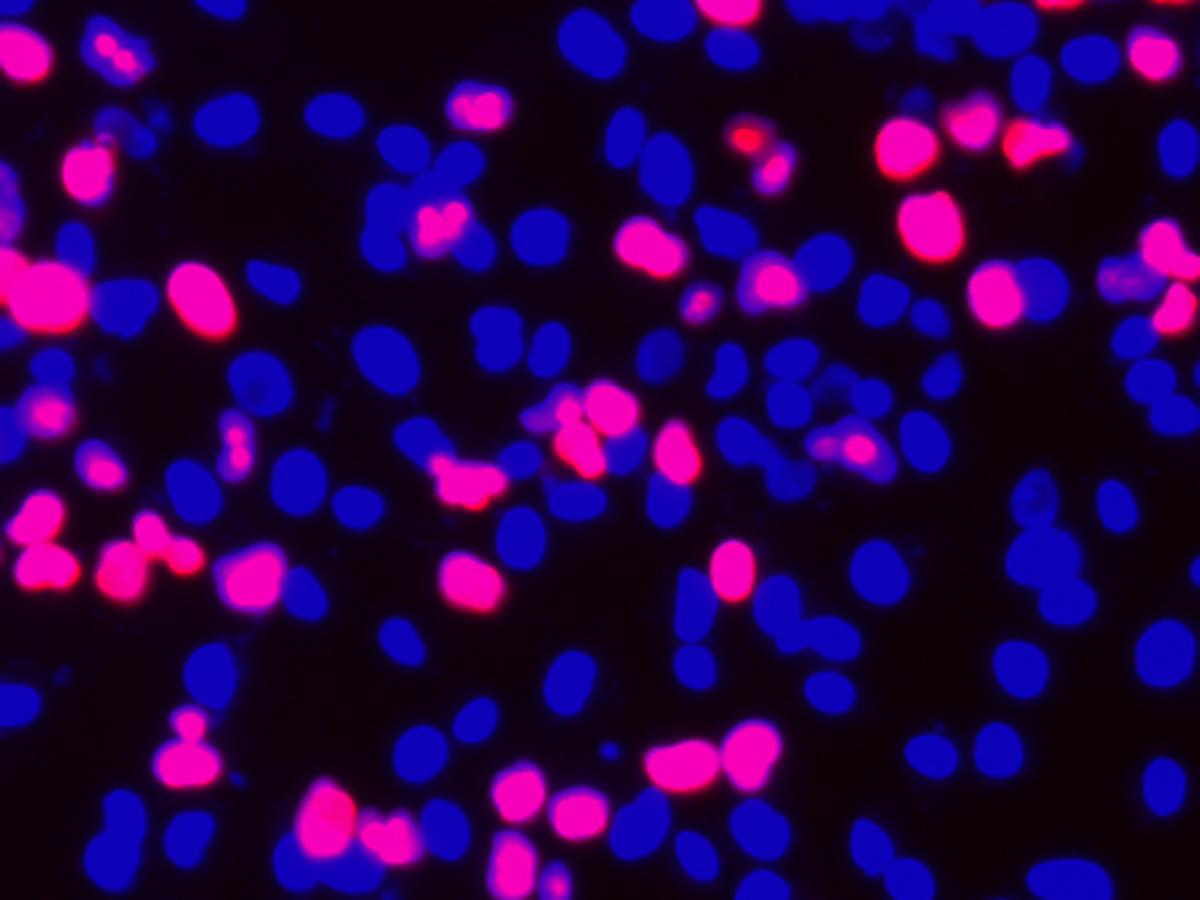

Supplement: Supplementary file 1 — Source Data for Figure 5G [file EMMM-14-e15618-s001.zip › Figure-5G-source-data/MCF7-SALL2-Ri#1-E2+-Ipatasertib-TUNEL.tif]

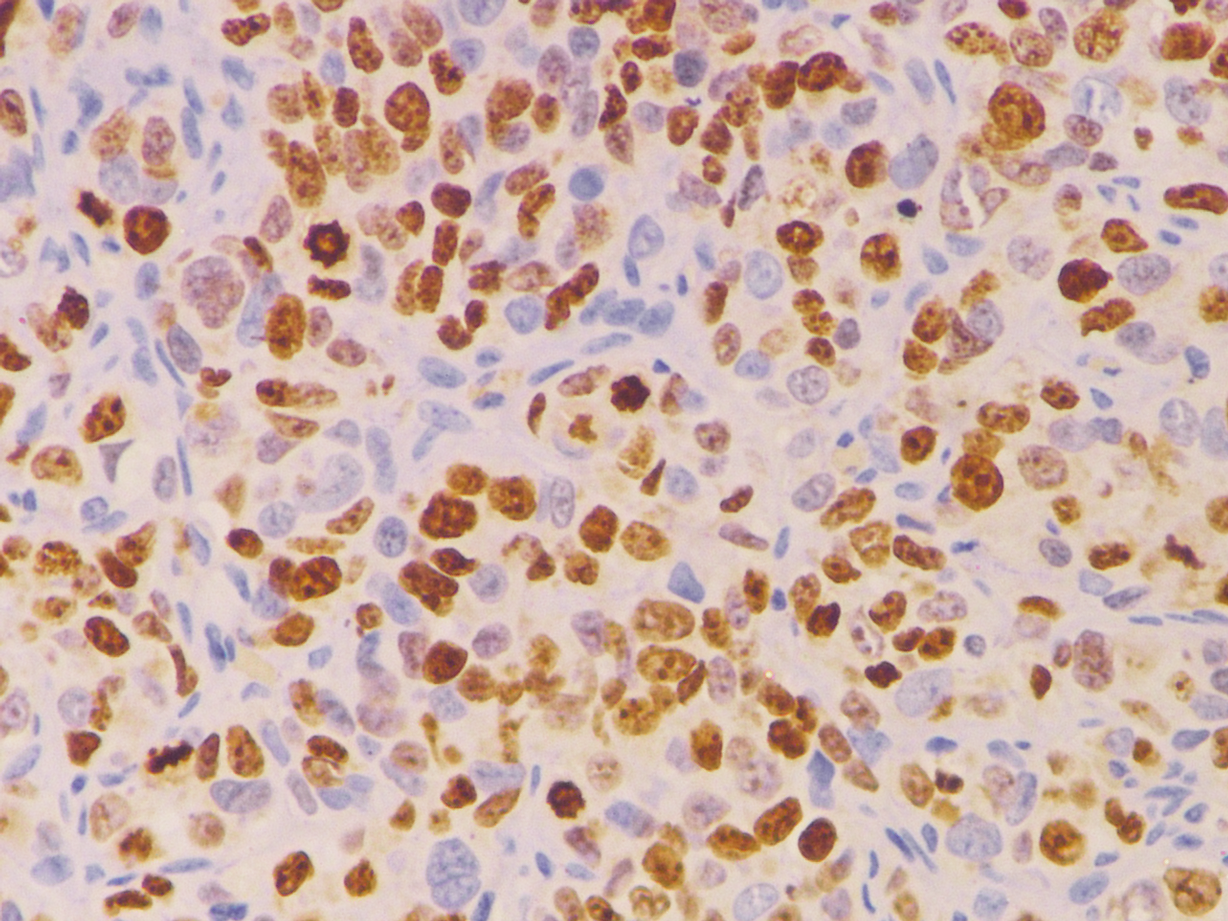

Supplement: Supplementary file 1 — Source Data for Figure 5G [file EMMM-14-e15618-s001.zip › Figure-5G-source-data/MCF7-SALL2-Ri#1-E2+-Veh-Ki67.tif]

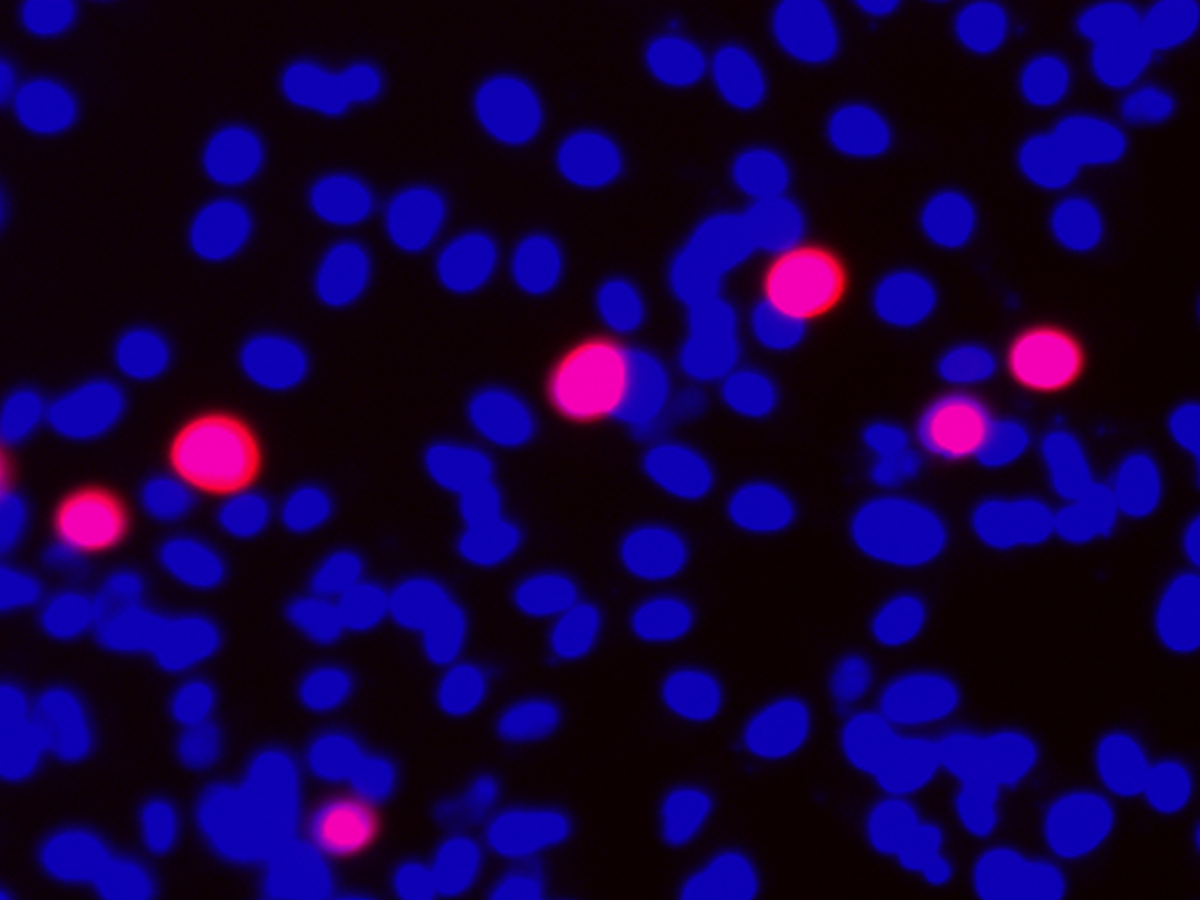

Supplement: Supplementary file 1 — Source Data for Figure 5G [file EMMM-14-e15618-s001.zip › Figure-5G-source-data/MCF7-SALL2-Ri#1-E2+-Veh-TUNEL.tif]
